# Stable isotope labelling kinetics of neurofilament light

**DOI:** 10.1093/braincomms/fcaf468

**Published:** 2025-12-17

**Authors:** Claire A Leckey, Tatiana A Giovannucci, John B Coulton, Yingxin He, Chihiro Sato, Nupur Ghoshal, Tharini Vignarajah, Zane Jaunmuktane, Nicolas R Barthélemy, Henrik Zetterberg, Donald L Elbert, Kevin Mills, Selina Wray, Randall J Bateman, Ross W Paterson

**Affiliations:** UCL Queen Square Institute of Neurology, Department of Neurodegenerative Disease, University College London, London WC1N 3BG, UK; UCL Great Ormond Street Institute of Child Health, Translational Mass Spectrometry Research Group, University College London, London WC1N 1EH, UK; UK Dementia Research Institute at University College London, London WC1N 6BT, UK; UCL Queen Square Institute of Neurology, Department of Neurodegenerative Disease, University College London, London WC1N 3BG, UK; UCL Great Ormond Street Institute of Child Health, Translational Mass Spectrometry Research Group, University College London, London WC1N 1EH, UK; UK Dementia Research Institute at University College London, London WC1N 6BT, UK; Department of Neurology, Washington University School of Medicine, St. Louis, MO 63110, USA; The Tracy Family SILQ Center, Washington University School of Medicine, St. Louis, MO 63110, USA; Department of Neurology, Washington University School of Medicine, St. Louis, MO 63110, USA; The Tracy Family SILQ Center, Washington University School of Medicine, St. Louis, MO 63110, USA; Department of Neurology, Washington University School of Medicine, St. Louis, MO 63110, USA; The Tracy Family SILQ Center, Washington University School of Medicine, St. Louis, MO 63110, USA; Department of Neurology, Washington University School of Medicine, St. Louis, MO 63110, USA; UCL Queen Square Institute of Neurology, Department of Neurodegenerative Disease, University College London, London WC1N 3BG, UK; UCL Queen Square Institute of Neurology, Department of Neurodegenerative Disease, University College London, London WC1N 3BG, UK; Queen Square Brain Bank for Neurological Disorders, Department of Clinical and Movement Neurosciences, University College London, London WC1N 1PJ, UK; Department of Neurology, Washington University School of Medicine, St. Louis, MO 63110, USA; The Tracy Family SILQ Center, Washington University School of Medicine, St. Louis, MO 63110, USA; UCL Queen Square Institute of Neurology, Department of Neurodegenerative Disease, University College London, London WC1N 3BG, UK; UK Dementia Research Institute at University College London, London WC1N 6BT, UK; Clinical Neurochemistry Laboratory, Sahlgrenska University Hospital, Mölndal SE-43180, Sweden; Department of Psychiatry and Neurochemistry, Institute of Neuroscience and Physiology, the Sahlgrenska Academy at the University of Gothenburg, Mölndal 40530, Sweden; Hong Kong Center for Neurodegenerative Diseases, Clear Water Bay, China; Wisconsin Alzheimer’s Disease Research Center, University of Wisconsin School of Medicine and Public Health, University of Wisconsin-Madison, Madison, WI 53792, USA; Department of Neurology, University of Washington, Seattle, WA 98195, USA; UCL Great Ormond Street Institute of Child Health, Translational Mass Spectrometry Research Group, University College London, London WC1N 1EH, UK; UCL Queen Square Institute of Neurology, Department of Neurodegenerative Disease, University College London, London WC1N 3BG, UK; Department of Neurology, Washington University School of Medicine, St. Louis, MO 63110, USA; The Tracy Family SILQ Center, Washington University School of Medicine, St. Louis, MO 63110, USA; UK Dementia Research Institute at University College London, London WC1N 6BT, UK; The Tracy Family SILQ Center, Washington University School of Medicine, St. Louis, MO 63110, USA; Dementia Research Centre, UCL Queen Square Institute of Neurology, London WC1N 3BG, UK; Darent Valley Hospital, Dartford DA2 8DA, UK

**Keywords:** neurofilament light chain, neurodegeneration, stable isotope labelling kinetics, human CNS, iPSC-derived neurons

## Abstract

This study provides the first quantification of neurofilament light chain (NfL) kinetics in the human CNS using stable isotope labelling kinetics. NfL is elevated in CSF and blood across a range of traumatic, inflammatory and neurodegenerative diseases of the CNS, and has been increasingly included in clinical trials as a secondary or exploratory outcome measure of target engagement. Interpreting trajectories of NfL post-treatment has been challenging, prompting a greater need and focus on understanding its pathophysiology. We set out to measure NfL kinetics in the human CNS using stable isotope labelling kinetics. In human neurons derived from induced pluripotent stem cells, we show that NfL turnover is relatively slow, comparable to other long-lived proteins such as tau. We detected a delay of 3 to 6 days in the release of NfL into the media, unexpected for a passively released protein and supporting that controlled mechanisms of release could contribute to the appearance of NfL in the extracellular milieu. We optimized the kinetic NfL assay to measure the turnover of NfL in the human CNS. Participants with diagnosed primary tauopathies (*n* = 10) were recruited to the Human CNS Tau Kinetics in Tauopathies study and a cohort of cognitively unimpaired or with mild cognitive impairment (Clinical Dementia Rating score ≤0.5; *n* = 22) to the Tau Stable Isotope Labelling Kinetics study. Patients with suspected normal pressure hydrocephalus (*n* = 3) and primary tauopathy cases (*n* = 3) were examined to assess labelling in the brain parenchyma and ventricular CSF. In brain tissue, isotopically labelled *in vivo* and sampled *ex-vivo* and *post-mortem*, NfL is rapidly labelled but remains stable 18 months after, indicating very slow turnover and likely incorporation into very stable NfL networks. In line with a controlled mechanism of release *in vivo*, appearance of labelled NfL in CSF was detectable between 53 and 162 days post-labelling, during which NfL labelling did not reach its peak, suggestive of a half-life in CSF >3 months. These findings support the interpretation that acute rises in CSF NfL concentration are likely to be related to passive release or CSF clearance failure. We also show that active but delayed release of newly translated NfL can contribute to the concentration of NfL in CSF, but this would not be expected for at least 8 weeks. Clinical trials using NfL as an outcome measure will benefit from substantially longer follow-up periods and isotopic labelling to understand the NfL response to therapeutic intervention.

## Teaser

Leckey *et al*. report novel measures of neurofilament light chain kinetics in the human central nervous system using stable isotope labelling. Translation occurs within hours, but appearance in cerebrospinal fluid takes ∼100 days. Labelled neurofilament light remains detectable in *post-mortem* brain tissue after 1.5 years, indicating slow turnover *in vivo*.

## Graphical Abstract

**Graphical Abstract:**
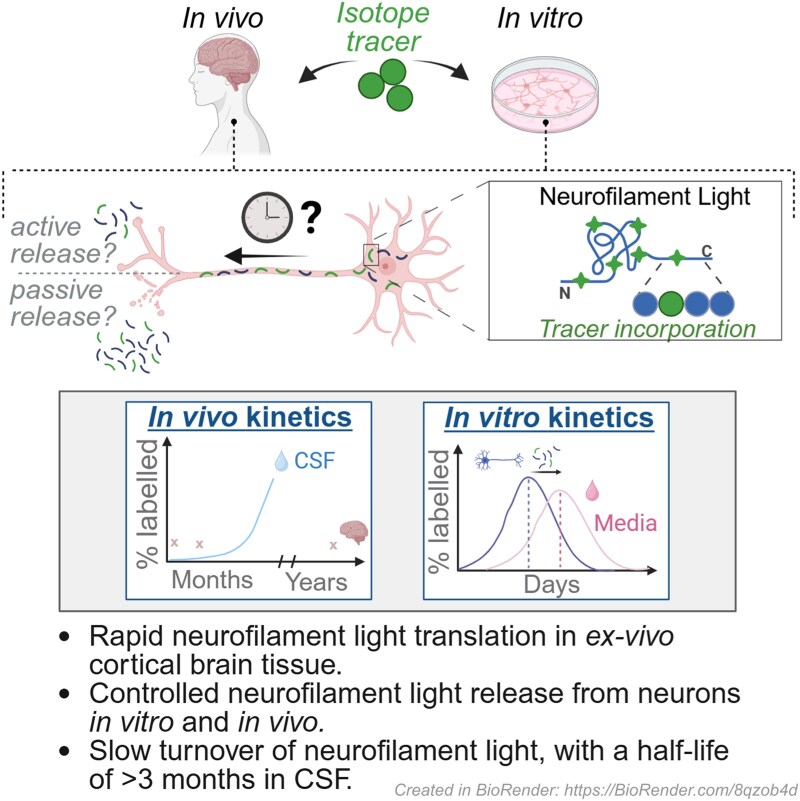

## Introduction

Neurofilament light chain (NfL) has emerged as a promising biomarker of neurodegeneration in blood and CSF.^1^ Elevated NfL has been observed across a wide range of neurological conditions.^2,3^ Remarkably, NfL levels dynamically reflect acute neural damage, rapidly increasing within 0–48 h following traumatic brain injury (TBI) and hypoxic brain injury,^4,5^ and acutely following clinical relapse in multiple sclerosis (MS).^6^ It can also fall in response to clinically successful disease modification.^7,8^ This dynamic behaviour makes NfL an attractive biomarker for disease monitoring and prognostication.

Yet, precise biological implications of elevated NfL levels remain unclear. NfL is a neuroaxonal protein that assembles into heteropolymers with neurofilament medium, heavy^9^ and alpha-internexin^10^ in the CNS to form the filament network that sustains axonal architecture,^11^ the distribution of organelles across axons^12^ and the stability of synaptic receptors.^13-15^ Possible explanations for the appearance of NfL in CSF include unregulated passive release from disrupted axons, active release through increased processing and secretion of NfL into the three major proteoforms,^16-18^ upregulated NfL synthesis or a combination of these mechanisms.

Determining the timing and magnitude of NfL responses is important for understanding when target engagement occurs and how to interpret changes in NfL levels during clinical trials. Furthermore, the disappearance of NfL from the CSF is also likely to be under the influence of several possible clearance mechanisms and routes, which may be disrupted in neurodegeneration or by the intrathecal delivery of therapies.

To address these open questions, we developed a Stable Isotope Labelling Kinetics (SILK) assay for NfL, a technique previously used to determine the kinetics of proteins related to neurodegeneration.^19-21^ NfL–SILK can be used to measure NfL kinetics in CSF and estimate the timing of NfL synthesis and release in humans.

## Materials and methods

This study was conducted in accordance with the Declaration of Helsinki and follows the Strengthening the Reporting of Observational Studies in Epidemiology (STROBE) reporting guidelines.

### Human CNS tau kinetics in tauopathies (TANGLES) study design

The NHS Health Research Authority, Research Ethics Committee London–Bloomsbury gave ethical approval for the University College London (UCL) TANGLES study (REC reference 18/LO/8601).

Participants who fulfilled clinical criteria for corticobasal degeneration (CBD^22^), progressive supranuclear palsy (PSP) and behavioural variant frontotemporal dementia (bvFTD; by confirmed genetic mutation in *MAPT*), and who had given prior consent for participation in research studies were recruited between 2019 and 2020 from specialist clinics at University College London Hospital and through Join Dementia Research.

All donors undergoing brain donation or their next of kin gave written informed consent at registration. Queen Square Brain Bank protocols and part of the study involving donated brain tissue have been approved by the NHS Health Research Authority, Ethics Committee London-Central (REC reference 23/LO/0044).

### UCL normal pressure hydrocephalus SILK study recruitment criteria

The UCL normal pressure hydrocephalus (NPH) SILK study was approved by the Bloomsbury ethics committee and all individuals provided informed written consent. Individuals with suspected idiopathic normal pressure hydrocephalus were recruited between 2019 and 2020 from the specialist hydrocephalus service at the National Hospital for Neurology and Neurosurgery, Queen Square (REC reference 19/LO/1256).

### WashU control and mild cognitive impairment SILK study recruitment criteria

This cohort is part of the Tau SILK study, approved by the Washington University IRB committee (Ref 201502091) and all individuals provided informed written consent. Individuals were recruited between 2015 and 2017 from the Volunteer for Health at Washington University St Louis as previously described.^20^ Individuals with clinical dementia rating (CDR) scores lower or equal to 0.5 were included and amyloid beta status was defined by amyloid PET with florbetapir ([F-18] AV-45) using a cut-off of 1.22 as previously described^20^ and if not available, by CSF amyloid beta 42:40 ratio measured by immunoprecipitation (IP) mass spectrometry.

### Differentiation of induced pluripotent stem cell into cortical neurons

Ctrl1 and Ctrl2 refer to the well-characterized SIGi1001-a-1 and RBi001-a, respectively, both available via Sigma Aldrich. Ctrl3 refers to a patient-derived cell line kindly shared by Dr Tilo Kunath.^23^

Induced pluripotent stem cells (iPSCs) were differentiated to cortical neurons using established protocols.^24,25^ Briefly, iPSCs at 100% confluence were subjected to neural induction using dual SMAD inhibition in N2B27 media (1 μM dorsomorphin and 10 μM SB431542, both TOCRIS). N2B27 media consists of 50% Dulbecco's Modified Eagle Medium-F12, 50% Neurobasal supplemented with 0.5× N2 supplement, 0.5× B27 supplement, 0.5× L-glutamine, 0.5× non-essential amino acids, 0.5× penicillin/streptomycin, insulin (25 U) and β-mercaptoethanol (1:1000). Cultures were passaged using Dispase. Progenitors underwent a final passage using Accutase at 35 DIV and neuronal maturation was performed in N2B27 media. Day 70 was taken as the starting point for *in vitro* NfL–SILK.

We characterized all cell lines for neuronal developmental markers by quantitative PCR and immunocytochemistry (Supplementary Methods and Supplementary Tables 1–3).

### NfL–SILK in iPSC-derived neurons

iPSC-derived neurons at 70 days *in-vitro* (DIV) were labelled with N2B27 media containing equal amounts of ^13^C_6_- and ^12^C-leucine [50% mol, 100% tracer-to-tracee ratio (TTR) media] for 24 days (full media change every three days), followed by culture in label-free N2B27 for 15–21 days.

#### Sample collection

Cells were washed once with phosphate buffered saline (PBS) and collected into 1.5 ml microfuge tubes using a cell scrapper, followed by centrifugation at 300×g for 10 min at 4°C. After removing PBS, cell pellets were snap frozen on dry ice and stored at −70°C until the entire experiment was completed. Conditioned media from a 12-well plate (1 ml/well) was pooled in 15 ml falcon tubes and centrifuged at 300×g for 10 min at room temperature to remove cell debris. Supernatant was transferred to 15 ml falcon tubes and stored at −7°C.

#### Intracellular NfL–SILK

Cells were lysed using radio-immunoprecipitation assay buffer (10 mM Tris-HCl pH 7.5, 140 mM NaCl, 0.5 mM EGTA, 1 mM EDTA, 1% Triton X-100, 0.1% sodium deoxycholate and 0.1% SDS) freshly supplemented with protease inhibitor (complete Mini, EDTA-free cocktail tablets, Roche). Cell membranes were mechanically disrupted using a syringe and sonicated using a sonicator (Ultrasonic Processor XL) for three cycles of 4 s at 40 amplification. Lysates were pre-cleared at 16 000×g for 10 min at 4°C and the soluble fraction (supernatant) collected in a separate LoBind (Eppendorf) tube.

**Figure 1:**
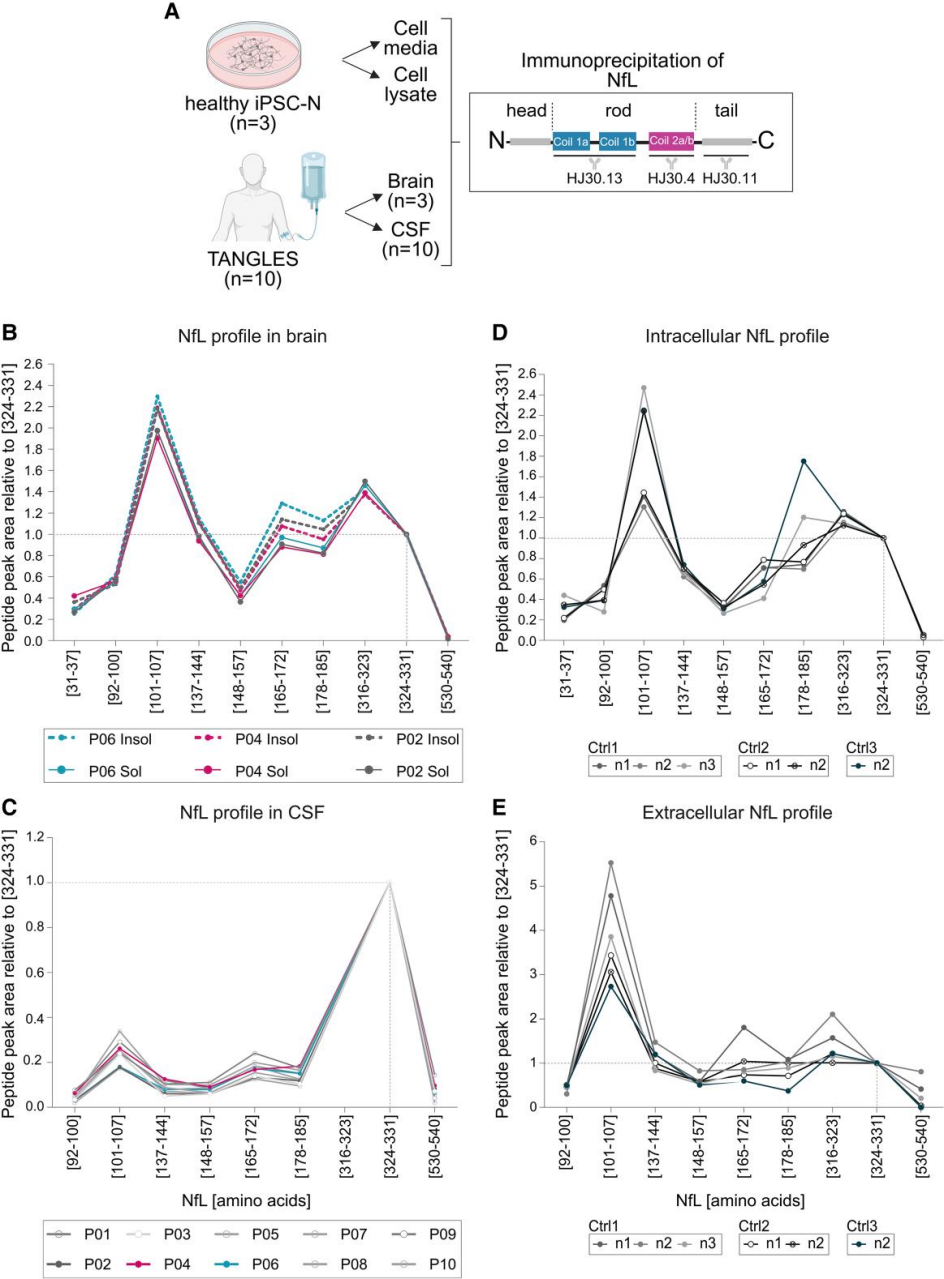
**NfL profiling *in vivo* and *in vitro*.** (**A**) Schematic of samples and processing. Created in BioRender https://BioRender.com/dxsbw52. (**B**) Brain recovery of NfL proteotypic peptides. Each peptide is indicated by its first- and last position in the canonical NfL amino acid sequence between brackets. *Post-mortem* cortical samples were processed into sarkosyl-soluble and insoluble fractions. Data from three individual samples is shown. (**C)** CSF recovery of NfL proteotypic peptides. Data from 10 participants is shown. (**D**) Intracellular recovery of NfL in iPSC-derived neurons from three control lines. (**E**) Extracellular recovery of NfL in conditioned media from iPSC-derived neurons from three control lines. Independent inductions shown (*n* = 3 Ctrl1; *n* = 2 Ctrl2; *n* = 1 Ctrl3). All peptide profiles are shown as peak areas relative to peptide (324–331), indicated by dashed lines. Abbreviations: CSF, cerebrospinal fluid; iPSC-N, induced-pluripotent stem cell–derived neurons; NfL, neurofilament light chain; Sol, soluble; Insol, insoluble; TANGLES, Human CNS Tau Kinetics in Tauopathies study.

Protein precipitation was performed with three volumes of ice-cold acetone for 16 h at −20°C. Precipitated protein pellets were resolubilized in 40 µL digest buffer [100 mM Tris-HCl pH 8, 6 M Urea, 2 M Thiourea, 2% (w/v) ASB-14] [spiked with 1 ng of a heavy labelled (^13^C, ^15^N -Arg/Lys) full-length recombinant NfL protein (61.5 kDa; human sequence) internal standard (Promise Proteomics, France) in the profiling experiments in Fig. 1] and 150 ng of yeast enolase as internal control. Proteins were reduced by incubation with 1,4-dithioerythritol (90 µg/sample) for 1 h with shaking before being alkylated by addition of iodoacetamide (216 µg/sample) and left to incubate in the dark for 1 h. Samples were diluted with addition of ultrapure grade water (331 µL) and digested with addition of MS grade Trypsin/Lys C (Promega) to a final concentration of 2 µg/ml for 16 h at 37°C with shaking. Trypsin was quenched by the addition of an equal volume of 0.2% formic acid (FA) in H_2_O. Resultant tryptic peptides of NfL were isolated and cleaned up via SPE (C18 Bond Elut, Agilent Technologies). Stationary phase was wetted with 70% acetonitrile (ACN), 0.1% FA (1 ml) and re-equilibrated with 0.1% FA (thrice 1 ml). NfL peptides were loaded to Toptips via centrifugation (500 rpm, 5 min) and washed by adding 0.1% FA (thrice 1 ml) and centrifugation. Peptides were eluted by adding 70% ACN, 0.1% FA (two additions of 250 μL) and centrifugation. Cleaned peptide extracts were concentrated by evaporation of eluent *in vacuo* and reconstituted in 40 μL 3% acetonitrile with 0.1% FA. 10 μL were typically used for each multiple reaction monitoring (MRM) NfL assay.

#### Extracellular NfL–SILK

400 µL of 25× master mix stock (25× NP-40, 125 mM guanidine hydrochloride, 25× protease inhibitor cocktail in PBS) were added to 10 ml of conditioned media and incubated under rotation at 4°C for 30 min. Samples were concentrated using 15 ml Amicon filters with a 10 kDa cut-off at 4500 rpm for 30 min at room temperature. This step concentrated the media down to 300–500 μL (approx. 20× concentration). *Immunoprecipitation:* concentrated media was incubated with mouse IgG2a-coupled M-270 Epoxy Dynabeads^TM^ for 1 h at 4°C to exclude unspecific bindings to the beads. In parallel, 90 µL M-270 Epoxy Dynabeads^TM^ coupled to anti-NfL HJ30.13, HJ30.4 and HJ30.11 antibodies, or mouse IgG as negative control (approx. 9 μg of antibodies/90 µL beads) were blocked using horse myoglobulin in PBS (1 mg/ml) for 1 h at 4°C. Concentrated, pre-cleared media was incubated with anti-NfL/anti-IgG coupled beads overnight at 4°C. Beads were then washed three times with 1 ml of 25 mM triethylammonium bicarbonate. NfL-coupled beads were suspended in 40 µL digest buffer (containing recombinant heavy labelled NfL in the profiling experiments in Fig. 1) and 75 ng yeast enolase for on-bead reduction, alkylation, digestion, SPE clean up and resuspension prior to ultra-performance liquid chromatography-tandem mass spectrometry (UPLC–MS/MS) analysis as described above.

### NfL–SILK in human subjects

#### UCL TANGLES SILK study

Briefly, individuals received a ^13^C_6_-leucine infusion intravenously at 4 mg/patient kg/h for 16 h. When infusion stopped, CSF was collected by lumbar puncture (LP) across five visits, which fell on the following number of days post-infusion: Day 4 (LP 1), Day 8 (LP 2), between Days 13 and 15 (LP 3), between Days 53 and 67 (LP 4) and between Days 112 and 162 (LP 5). CSF was centrifuged at 1000×g for 10 min at 4°C, aliquoted into protein low-bind tubes (MAXYmum Recovery, Axygen) and stored at −80°C within 1 h of collection.

#### Normal pressure hydrocephalus SILK study

Individuals received a ^13^C_6_-leucine infusion intravenously at 3 mg/patient kg/h for 10 min, then 2 mg/patient kg/h for between 3 and 16 h prior to either a diagnostic lumbar drain insertion or ventriculoperitoneal (VP) shunt insertion. A full thickness cortical biopsy was collected during VP shunt insertion as previously described.^26^

The enrichment of labelled leucine in plasma was measured as described in the Supplementary Methods section (Supplementary Table 4).

#### WashU tau SILK study

Individuals received a ^13^C_6_-leucine infusion intravenously at 4 mg/patient kg/h for 16 h using an identical protocol to the UCL TANGLES SILK study.

##### Peptide-level immunoprecipitation–MS/MS of NfL in CSF

CSF was prepared and analysed by peptide IP-MS/MS as previously described,^17^ but with the following adaptation: CSF sample volume increased to 1000 µL prior to spiking with 1 ng of a heavy labelled [^13^C, ^15^N-Arg/Lys] full-length recombinant NfL protein (61.5 kDa; human sequence) internal standard (Promise Proteomics, France). TANGLES samples were additionally prepared by protein-level IP (see below).

##### TANGLES protein-level IP-MS/MS

CSF samples were prepared using the protein-level IP method previously described,^17^ but with a few alterations. CSF sample volume was increased to 1000 µL and spiked with 1 ng heavy-labelled full-length recombinant NfL internal standard (Promise Proteomics, France) and 50 µL of Master Mix to provide the following final concentrations in sample (0.5% NP-40, 2.5 mM guanidine and 1× Roche cOmplete Protease Inhibitor). M-270 Epoxy Dynabeads^TM^ were coupled to anti-NfL HJ30.13, HJ30.4 and HJ30.11 antibodies as previously described,^17^ and 30 µL of the coupled-bead slurry was added to each sample and incubated with rotatory shaking for 2 h at room temperature (20°C). IP proceeded as previously described,^17^ however on-bead tryptic digestion was performed as follows: 40 μL digest buffer (8 M urea, 200 mM Tris HCl, pH 8) was added to NfL-antibody-epoxy beads and incubated for at least 30 min. Reduction and alkylation was performed with dithioerythritol (90 μg/sample) at room temperature with shaking at 1500 rpm for 1 h, followed by incubation with iodoacetamide (216 μg/sample) in the dark and at room temperature with shaking at 1500 rpm for 50 min. Samples were diluted with high-performance LC-grade ultrapure water prior to addition of MS-grade Trypsin–Lys-C to a final concentration of 2 µg/ml, and samples were digested at 37°C for 16 h. Digested bead samples then underwent C18 solid phase extraction prior to UPLC-MS/MS analysis as described above for intracellular SILK.

##### IP-MS/MS of NfL from soluble and insoluble fractions of post-mortem brain tissue

Brain samples from frontal cortex were processed following protocols from Mukherjee *et al.*^27^ Briefly, 0.27–0.57 gram of grey matter from frontal cortex tissue was homogenized in 10 volumes (v/w) of lysis buffer (10 mM Tris–HCl, pH 7.4, 800 mM NaCl, 1 mM EDTA, 2 mM DTT, 10% sucrose) using a probe sonicator (TissueRuptor, QIAGEN) on ice until completely homogenized. The starting brain homogenate was centrifuged at 16 000×g for 20 min at 4°C. The crude supernatant was treated with *N*-lauroylsarcosinate (‘sarkosyl’, 1% [w/v] final concentration) and shaken at room temperature for 1 h. The supernatant was then centrifuged at 100 000×g for 1 h at 4°C. The sarkosyl-insoluble pellet was resuspended in 300 μL washing buffer (10 mM Tris–HCl, pH 7.4, 800 mM NaCl, 5 mM EDTA, 2 mM DTT, 10% sucrose) and centrifuged at 16 000×g for 30 min at 4°C. After centrifugation, the supernatant was further centrifuged at 100 000×g (Optima MAX, Beckman Coulter) for 1 h at 4°C. The supernatant was kept in protein LoBind tubes (Eppendorf) as the sarkosyl-soluble fraction for IP of NfL. The pellet was resolubilized in 70% FA using a probe sonicator and dried down by evaporation of eluent *in vacuo*.

For IP of NfL from the insoluble fraction, lyophilized proteins were resuspended in a small amount of 70% FA and neutralized with Tris-HCl pH 11. Sarkosyl soluble and insoluble fractions at 1 mg/ml in 500 µl were used for IP, adding Triton-X100 to a final concentration of 0.1%. IP is further described in ‘Extracellular NfL-SILK’ (Supplementary Methods).

An unlabelled brain sample from the frontal cortex of a *post-mortem* non-degenerative case (female, 9th decade of life) was processed to assess the technical noise of the NfL–SILK method.

### NfL–SILK quantitation

UPLC-MS/MS analysis was performed as previously described,^19^ with adaptions to monitor SILK labelled peptides (see Supplementary Table 4). Acquired data were imported into Skyline software (MacCoss Lab, University of Washington). Integrated peak areas were used to calculate the TTR, fractional synthesis rate (FSR), fractional clearance rate (FCR) and half-life of NfL following methods described in the Supplementary Methods section.

### Statistical analysis

Statistical analyses were performed using GraphPad Prism v10.1.2. To test for Gaussian distribution, the Shapiro–Wilk normality test was used. If the normality test was passed, data were analysed by Student’s unpaired *t*-test (two groups) or by ANOVA, otherwise. Statistical analysis of data when normality could not be assumed (did not pass normality test or *n* numbers were too low) was performed using the nonparametric Mann–Whitney test (two groups) or Kruskal–Wallis test for multiple comparisons, with Dunn’s or Tukey’s test to adjust for multiple comparisons.

## Results

### NfL profiling in brain, CSF and iPSC-derived neurons

We employed a mass spectrometry assay together with IP to characterize NfL, including potential truncated species, in brain and CSF from a cohort of individuals with primary tauopathies (TANGLES cohort). NfL was immunopurified from lumbar CSF (*n* = 10), as well as from three brain samples from the same cohort and a non-degenerative case donated *post-mortem*. Brain samples were biochemically fractionated into sarkosyl-soluble and insoluble fractions to determine the solubility profile of NfL.^28^ Details of the cohort can be found in Table 1.

**Table 1:** Characteristics of the neurodegenerative cohorts included in this study

Participant ID	Clinical diagnosis	Path-confirmed diagnosis	Decade of life	Sex (F/M)^a^	CSF NfL (pg/ml)^b^	Brain donation	Time from labelling to donation (days)	Time post-labelling when labelled NfL was first detected (days)
TANGLES SILK^c^ study								
P01	bvFTD^d^	NA^e^	5th	M	1796	NA	NA	123 (LP^f^ 5)
P02	CBD^g^	CBD	7th	F	3378	*Post-mortem*	574	None
P03	PSP^h^	NA	7th	M	1097		NA	None
P04	bvFTD	bvFTD (autosomal dominant MAPT mutation)	7th	M	N/A	*Post-mortem*	1344	None
P05	CBS^i^	NA	8th	F	3991	NA	NA	112 (LP 5)
P06	CBS	Pick’s disease	7th	F	4404	*Post-mortem*	1525	120 (LP 5)
P07	PSP	NA	8th	M	544	NA	NA	None
P08	PSP	NA	7th	M	1687	NA	NA	53 (LP 4)
P09	PSP	NA	8th	M	3244	NA	NA	None
P10	PSP	NA	7th	M	3672	NA	NA	60 (LP 4)
NPH^j^ SILK study								
N01	iNPH^k^	NA	9th	M	935	*Ex-vivo*	4 h	None
N02	iNPH	NA	8th	M	N/A^l^	*Ex-vivo*	44	None
N03	iNPH	NA	7th	M	N/A	NA	128	128

^a^F/M: Female/male. ^b^CSF NfL (pg/ml): The static NfL values were measured by SIMOA (Quanterix). ^c^TANGLES SILK: Human CNS Tau Kinetics in Tauopathies (TANGLES) Stable Isotope Labelling Kinetics (SILK). ^d^bvFTD: behavioural variant frontotemporal dementia. ^e^NA: Not applicable. ^f^LP: Lumbar puncture. ^g^CBD: Corticobasal degeneration. ^h^PSP: Supranuclear palsy. ^i^CBS: Corticobasal syndrome. ^j^NPH: Normal pressure hydrocephalus. ^k^iNPH: Idiopathic normal pressure hydrocephalus. ^l^N/A: Not available.

We used a combination of three custom monoclonal antibodies targeting discrete regions across the NfL sequence (Fig. 1A) and normalized peptide abundances to the Coil 2B peptide (residues [324–331]) to aid comparisons between compartments. The predominant form of NfL in CSF consisted of proteoforms containing mostly the Coil 2B domain, whilst in brain tissue higher relative abundances of peptides in the head, Coil 1A and Coil 1B domains were observed (Fig. 1B and C; Supplementary Fig. 1). These results are consistent with previous studies describing full length NfL recovery in brain, but truncated forms in CSF.^16,17^ We found that NfL was relatively more abundant in the soluble brain fraction but was also recovered in smaller amounts in the insoluble fractions, largely maintaining the same peptide profile (Fig. 1B; Supplementary Fig. 1). Comparison to a cognitively unimpaired brain showed that the proportion of NfL in the insoluble fraction was 4.2-fold higher in P02 and 2.5–2.2-fold higher in P04 and P06 (Supplementary Fig. 1). This is in line with previous mass spectrometry-based reports identifying NfL peptides in insoluble protein inclusions and neurofibrillary tangles.^29,30^ The N-terminal domain of NfL is a region of low complexity and target of post-translational modifications including phosphorylation.^31^ Our assay included a peptide in the N-terminal domain of NfL [residues (31–37)], which was recovered from brain but largely reduced in CSF (Fig. 1B and C) and was more abundant in the control than in the tauopathy brains (Supplementary Fig. 1).

Neurons from human iPSC are a translational model uniquely placed to examine neural function and model disease processes *in vitro*. We compared NfL profiles from cell extracts and conditioned media to human intracellular (brain) and extracellular (CSF) compartments. Three iPSC lines derived from cognitively normal controls at the time of biopsy were differentiated into cortical neurons^25^ in several independent batches (characterization of these cells can be found in Supplementary Fig. 2). We found the N-terminal peptide [residues (31–37)] in cell lysates and brain, which was largely reduced in cell media and CSF. Contrary to CSF, but similar to brain, cell media and lysates were rich in Coil 1A peptides [residues (101–107)]. C-terminal tail peptide recovery was low in all compartments. Coil 2B peptides, the most abundant in CSF across neurodegenerative conditions^17^ and the most widely measured domain of the protein as a clinical and research biomarker, were recovered from all compartments *in vivo* and *in vitro*.

### NfL kinetics in iPSC-derived cortical neurons

**Figure 2:**
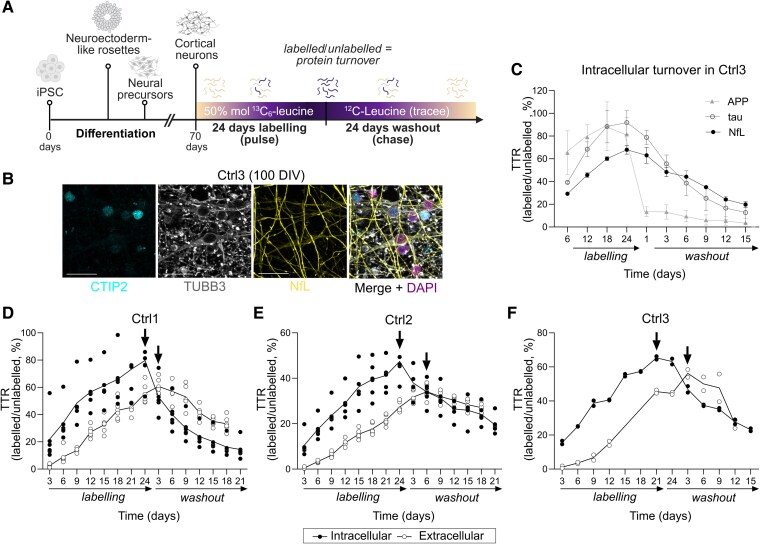
**NfL kinetics in iPSC-derived neurons from three non-degenerative donors.** (**A**) Schematic summarizing the experimental set up. Created in BioRender https://BioRender.com/d8e1bab. (**B**) Representative confocal image (maximal projection) of Ctrl3 neurons at 100 DIV stained for the pan neuronal marker Beta III tubulin (TUBB3), the deep-layer neuronal marker COUP-TF-interacting protein 2 (CTIP2) and neurofilament light chain protein (NfL). DAPI was used as nuclear counterstain. Scale bars = 20 µm. (**C**) Kinetic profiles of NfL, tau and APP obtained in Ctrl3 cells. Results are displayed as mean ± SD of three independent inductions (APP, tau) and two independent inductions (NfL) for a specific proteotypic peptide per protein [for NfL, peptide (137–144); for APP and tau see Supplementary Table 7]. (**D**) Ctrl1 results (*n* = 3–4 independent inductions). (**E)** Ctrl2 results (*n* = 2 independent inductions). (**F)** Ctrl3 intracellular results (*n* = 2 independent inductions). Datapoints represent the mean TTR of each peptide (*n* = 6) at a given timepoint from all inductions. Indicated with arrows is the time-to-peak of each intra- and extracellular kinetic curves. To see the kinetic profile of each independent peptide, please refer to Supplementary Fig. 4. Abbreviations: DIV, days-in-vitro; iPSC, induced-pluripotent stem cells; TTR, tracer-to-tracee ratio.

To study NfL kinetics *in vitro*, we adapted the mass spectrometry method for the detection of isotopically labelled leucine incorporation into six proteotypic peptides (namely, unique to NfL) following a SILK paradigm (Fig. 2A; Supplementary Table 5) using three iPSC lines derived from non-degenerative controls at the time of biopsy (Supplementary Table 1).

NfL intracellular turnover was relatively slow in all three lines (Fig. 2D–F and Supplementary Fig. 3), with a similar half-life between control lines (5.06 ± 0.96 days in Ctrl1 cells; 6.95 ± 2.79 days in Ctrl2; see Table 2 and Supplementary Table 6). Side-by-side comparison of the turnover of NfL, tau and amyloid precursor protein (APP) in the same line (Ctrl3) showed the capability of the NfL–SILK *in vitro* approach to capture the kinetics of fast- and slow turnover proteins (Fig. 2C and Supplementary Table 7). Cell integrity remained stable during the experiment (Supplementary Fig. 3). There were no differences between the six individual peptide’s half-lives (Ctrl1 cells; *H*(5) = 7.305, *P* = ns) (Table 2; Supplementary Fig. 4). For all the peptides monitored, we detected a delay in appearance of labelled NfL in the media, reflected in a delay of 3 to 6 days to achieve maximum labelling (‘time-to-peak’, a delay of 3 days in Ctrl1 cells and of 6 days in Ctrl2 and Ctrl3). This is consistent with previous findings in tau SILK^20^ and might be the result of active transport from intra- to extracellular as opposed to exclusive passive release from dying neurons (where no delay would be expected). Clearance rates were much higher in cell lysates (mean FCR 3.67 ± 0.51) than media (mean FCR 1.92 ± 0.48), consistent with a lack of clearance mechanisms from the media in this 2D-*in vitro* system and suggesting that there is limited degradation/proteolysis of NfL in neuronal conditioned media (Supplementary Table 8).

**Table 2:** Kinetic measurements in iPSC-derived neuron cell lysates

HALF-LIFE (Intracellular)				
		Ctrl1	Ctrl2	Ctrl3
Peptide	Amino acids^a^	Mean half-life (d^b^) ± SD^c^	Mean half-life (d)	Mean half-life (d)
AQLQDLNDR	92–100	5.79 ± 1.22	6.78	NC
ALYEQEIR	137–144	4.01 ± 1.10	38.29*^d^	NC^e^
LAAEDATNEK	148–157	3.91 ± 1.37	18.35*	7.76
FTVLTESAAK	284–293	5.48 ± 0.08	9.04	NC
TLEIEACR	316–323	6.25 ± 2.39	15.53*	NC
GMNEALEK	324–331	4.93 ± 0.62	4.53	NC
	Mean all	**5.06** ± 0.96	**6.95** ± 2.79	**7.76**

FRACTIONAL SYNTHESIS RATE (Intracellular)				
Peptide	Amino acids	Mean FSR^f^ (%/d) ± SD	Mean FSR (%/d) ± SD	Mean FSR (%/d) ± SD
AQLQDLNDR	92–100	2.16 ± 0.58	1.37 ± 1.48	NC
ALYEQEIR	137–144	3.42 ± 0.78	1.77 ± 1.65	2.38 ± 0.21
LAAEDATNEK	148–157	3.63 ± 1.10	1.74 ± 1.22	2.37 ± 0.00
FTVLTESAAK	284–293	2.86 ± 0.10	2.03 ± 2.06	NC
TLEIEACR	316–323	3.08 ± 1.49	1.70 ± 1.30	NC
GMNEALEK	324–331	2.51 ± 0.88	1.91 ± 1.41	NC
	Mean all	**2.94** ± 0.55	**1.75** ± 0.20	**2.37** ± 0.1

FRACTIONAL CLEARANCE RATE (Intracellular)				
Peptide	Amino acids	Mean FCR^g^ (%/d) ± SD	Mean FCR (%/d) ± SD	Mean FCR (%/d) ± SD
AQLQDLNDR	92–100	8.97 ± 2.15	3.51 ± 1.48	NC
ALYEQEIR	137–144	8.19 ± 1.66	3.93 ± 1.65	9.31 ± 1.51
LAAEDATNEK	148–157	7.69 ± 0.43	3.88 ± 1.22	8.15 ± 0.27
FTVLTESAAK	284–293	7.62 ± 0.35	4.39 ± 2.06	NC
TLEIEACR	316–323	7.89 ± 1.27	3.43 ± 1.30	NC
GMNEALEK	324–331	5.73 ± 0.31	2.89 ± 1.41	NC
	Mean all	**7.68** ± 1.08	**3.67** ± 0.51	**8.73** ± 0.82

^a^Amino acids in the consensus sequence of NfL as per UniProt entry P07196. ^b^d: days. The half-life estimates were calculated fitting the data into a one-phase decay model. ^c^SD: Standard deviation. ^d^*: Half-life measurements marked with an asterisk (*) were deemed unreliable (the model could not report a double-sided confidence interval) and were not included in the calculation of the average. ^e^NC: Not captured (chromatography peaks did not pass quality control checks and were not included in the analysis). ^f^FSR: Fractional synthesis rate (represented as percentage per day ± SD). ^g^FCR: Fractional clearance rate (represented as percentage per day ± SD).

### NfL kinetics in humans

**Figure 3:**
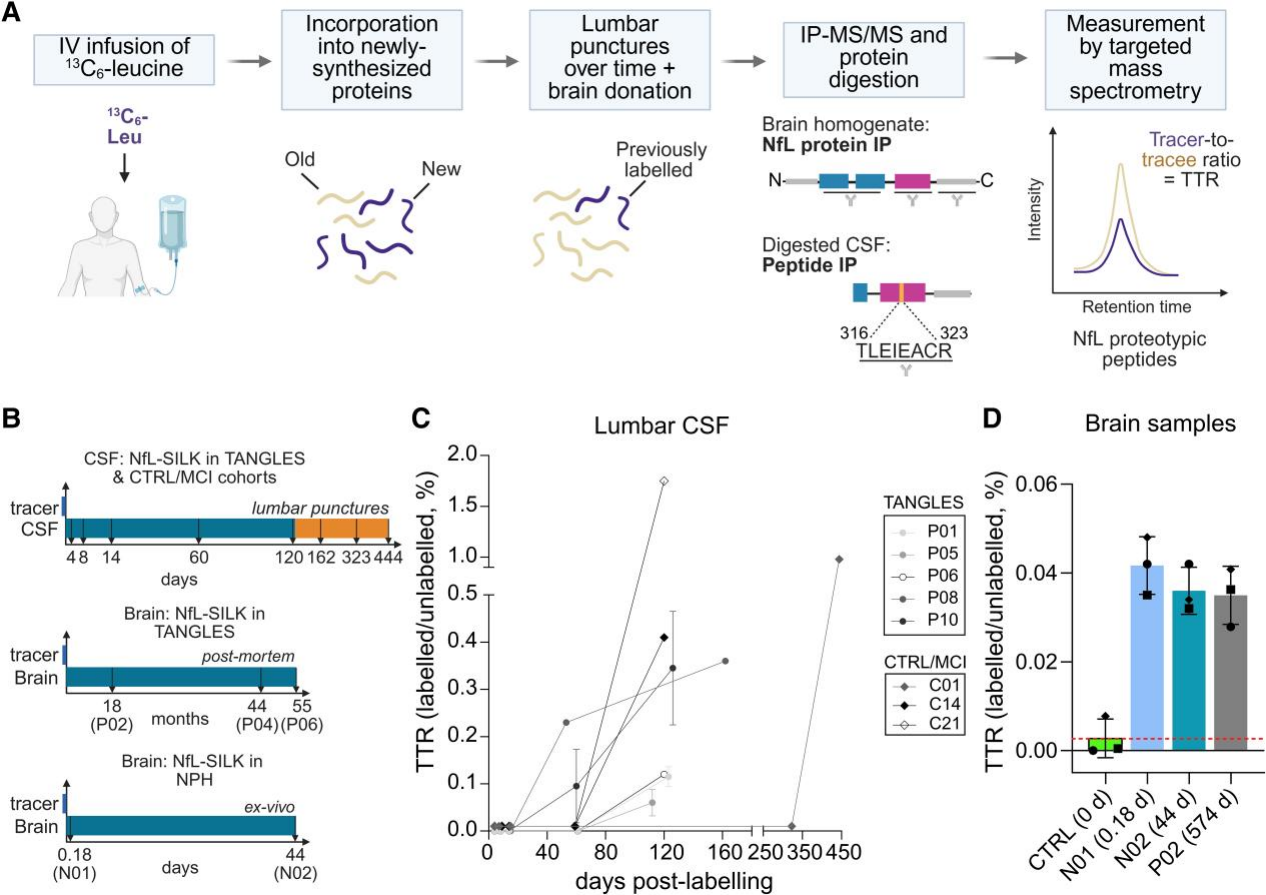
**NfL kinetics in the human CNS.** (**A**) Overview of the NfL SILK analysis pipeline *in vivo*. Created in BioRender https://BioRender.com/3x7d2jm. (**B**) Schematic summarizing the collection of samples included in the analysis. Lumbar punctures for both TANGLES (participants P01–P10) and the WashU Control and MCI study (participants C01–C22) were scheduled between 4- and 120-days post-labelling. However, some samples from the WashU cohort had extended collection timepoints (marked in orange) due to rescheduled visits because of the COVID-19 pandemic. Note that time axes are not drawn at scale. (**C**) NfL kinetic curves detected in CSF from TANGLES and the WashU Control and MCI study (‘CTRL/MCI’) participants. (**D**) ^13^C_6_-leucine incorporation into NfL in brain tissue. Labelled NfL in brain tissue donated at 4 h (N01), 44 days (N02) and 574 days (P02) post-labelling compared to baseline abundance of ^13^C_6_-labelled NfL in a non-labelled control brain. Data are depicted as mean ± SD of three NfL proteotypic peptides: (148–157), depicted with a squared symbol; (178–185), depicted with a diamond-shaped symbol; and (324–331), depicted with a round symbol. Abbreviations: CSF, cerebrospinal fluid; CTRL, control; d, days; IP-MS/MS, immunoprecipitation—tandem mass spectrometry; Leu, Leucine; MCI, mild-cognitive impairment; NfL, neurofilament light chain; NPH, normal pressure hydrocephalus; SILK, stable isotope labelling kinetics; TANGLES, Human CNS Tau Kinetics in Tauopathies study; TTR, tracer-to-tracee ratio; WashU, Washington University in St Louis.

To capture NfL dynamics *in vivo*, we analysed CSF and donated brain tissue from study participants from three neurodegenerative SILK cohorts: TANGLES (CSF and *post-mortem* brain in primary tauopathies), normal pressure hydrocephalus (NPH) SILK (CSF and *ex vivo* brain tissue from NPH patients), both summarized in Table 1; and a SILK cohort of cognitively normal individuals or with mild cognitive impairment (MCI; CDR ≤ 0.5), summarized in Table 3. Having established a greater enrichment of Coil 2B peptides in CSF during profiling (Fig. 1C), label incorporation into NfL was measured using a peptide IP-MS/MS approach for Coil 2B peptide TLEIEACR (Fig. 3A), while a full protein IP-MS/MS approach was used for brain tissue (Fig. 3A) due to its more uniform NfL profile distribution across the protein (Fig. 1B).

**Table 3:** Characteristics of the Wash U control/mild cognitive impairment cohort included in this study

Participant ID	CDR^a^	CSF Aβ42:40 ratio^b^	Amyloid PET AV45 (SUVR)^c^	Decade of life	Sex (F/M)^d^	NfL in CSF (pg/ml)^e^	Time post-labelling when labelled NfL is first detected (days)
C01	0.5	NA^f^	NA	7th	F	315	444 (LP^g^ 5)
C02	0	0.139	1.234	6th	F	309	None
C03	0	0.112	NA	6th	M	195	None
C04	0	0.194	1.017	7th	M	822	None
C05	0.5	0.106	NA	7th	M	644	None
C06	0.5	0.086	1.164	7th	M	851	None
C07	0.5	0.119	NA	7th	F	2192	None
C08	0	0.196	NA	7th	M	1313	None
C09	0	0.198	NA	6th	F	1171	None
C10	0.5	0.099	NA	7th	M	548	None
C11	0	0.182	1.107	8th	M	397	None
C12	0	0.207	1.102	8th	F	695	None
C13	0	0.241	1.196	7th	F	489	None
C14	0	0.112	1.360	6th	M	1222	120 (LP 5)
C15	0.5	0.201	0.949	7th	F	715	None
C16	0	0.183	1.160	7th	F	398	None
C17	0	0.143	1.195	7th	F	807	None
C18	0.5	0.112	1.856	8th	F	604	None
C19	0.5	0.162	NA	7th	F	501	None
C20	0.5	0.098	1.811	9th	F	997	None
C21	0	0.103	1.239	8th	F	684	120 (LP 5)
C22	0	0.172	1.106	7th	M	577	None

^a^CDR: Clinical dementia rating. ^b^CSF Aβ42:40 ratio: Amyloid-β (Aβ) 42:40 ratio in cerebrospinal fluid (CSF). ^c^Amyloid PET AV45 (SUVR): Standardized uptake value ratio (SUVR). The cut-off for amyloid positivity was set to 1.22 (see Materials and methods). ^d^F/M: Female/male. ^e^CSF NfL (pg/ml): The static NfL values were quantitated by IP-MS/MS using the known concentration of NfL internal standard spiked into each sample as a reference. ^f^NA: Not available. ^g^LP: Lumbar puncture.

In CSF, labelled NfL was detected in five out of nine (56%) TANGLES study participants, with plotted kinetic curves suggesting NfL turnover to be very slow, with low label incorporation (0.04–0.36%) observed by the end of the chase (Fig. 3B and C; Supplementary Figs 5 and 6). We then validated these findings in an independent cohort of cognitively normal individuals or MCI with low baseline NfL levels and extended chase periods (Table 3), where labelled NfL was detected in 3/22 (13.6%) participants (Fig. 3B and C; Supplementary Fig. 7). Ventricular CSF was collected and analysed from one individual with suspected NPH during VP shunt insertion, with 0.07% label incorporation detected 128 days post-labelling. Altogether, these data suggest low *in vivo* turnover of NfL in both ventricular- and lumbar CSF compartments (Supplementary Fig. 8).

For determining NfL turnover in brain, *ex vivo* tissue was collected during VP shunting in two individuals with suspected NPH at 4.3 h and 44 days post-labelling, while donated *post-mortem* tissue was analysed at substantially longer timepoints post-labelling (18–55 months) from the TANGLES cohort (Fig. 3B). Brain tissue was homogenized and fractionated before NfL was enriched by protein IP-MS/MS to quantitate labelled NfL ratios in sarkosyl-soluble and sarkosyl-insoluble fractions. Due to the ethical unfeasibility of obtaining brain tissue from a single individual at multiple timepoints for a kinetic curve, NfL SILK data in brain tissue from three individuals were plotted as NfL TTR (in the detergent-soluble fraction) versus time of collection post-labelling (Fig. 3D). Combined, the data show label incorporation into NfL at 0.03–0.05% from both cohorts up to 549 days post-label, which was the longest timepoint that could be reliably analysed. Incorporation of ^13^C_6_-leucine into the three peptides analysed was above and distinguishable from a non-labelled brain, representative of the isotopic natural abundance level. Overall, the data indicate that NfL translation in the brain is detectable within hours of tracer infusion, and that newly synthesized NfL remains metabolically stable over months in the population studied.

## Discussion

We provide the first quantitation of NfL kinetics in the human CNS and neurons using SILK. We show that intracellular NfL translation occurs within 4 h in *ex vivo* human brain, but detection of labelled (newly synthesized) NfL in CSF is first observed around 53 days post-labelling. Together, this data suggests that NfL is translated rapidly in brain, while its release into CSF is very slow, with no peak of label incorporation or clearance captured during the study’s 5.4-month chase period. Labelled (new) NfL in brain can be detected at stable levels in cortical brain tissue as long as 1.5 years after SILK labelling. This is consistent with data in animal models suggesting there is a relatively small finite source of stable NfL in the human CNS and turnover of NfL is extremely slow.^32,33^

To study brain–CSF NfL dynamics, we analysed CSF samples from the TANGLES cohort, the control and MCI cohorts, and a single individual with NPH together with donated *post-mortem* brain tissue from three TANGLES participants and with *ex vivo* tissue from two participants of the NPH SILK cohort.

Analysis of labelled NfL in frontal cortex samples showed that NfL is rapidly translated, within hours of labelling, but remains in the stable cytoskeletal lattice from hours to months. Labelled NfL levels in sarkosyl-soluble fractions from *ex vivo* tissue samples taken at 4.3 h and 44 days post-labelling (0.042–0.033% TTR) and at 549 days post-labelling in *post-mortem* brain tissue (0.033%) remained stable. Without access to samples between 44 and 549 days, we cannot discard dynamic changes during that period—however, the stable retention of newly synthesized NfL within neurons is supported by the delayed detection of labelled NfL in CSF; first detected between 53 and 162 days post-labelling, and with only the start of the kinetic curve captured during the 5.4-month study period.

In humans, dynamic responses of NfL have been studied longitudinally by measuring changes in static CSF, plasma and/or serum NfL concentrations. This has been particularly instructive in scenarios where the steady state of NfL is challenged, e.g. acute brain injury (TBI, neuroinflammation or stroke). In TBI, rises are seen in CSF and plasma within 7–10 days and fall to normal background levels within 120–180 days.^4,34^ This has been interpreted as reflecting passive release of established reservoirs of axonal NfL rather than reflecting new NfL synthesis. We do not have access to SILK labelled individuals undergoing acute brain injury, but the timing of labelled NfL appearance in our cohort supports this conclusion.

Clinical trials of disease-modifying therapies have brought sharp focus on the interpretation of NfL, particularly when used as a biomarker of therapeutic effect. In Alzheimer’s disease, plasma NfL levels were monitored in both the TRAILBLAZER-ALZ randomized clinical trial for Donanemab and the phase 3 of the Clarity AD trial for lecanemab, with plasma NfL levels showing no significant changes or improvements when the treatment groups were compared to placebo.^35,36^ Interestingly, in the TRAILBLAZER-ALZ trial, plasma NfL was the only significant biomarker whose change from baseline negatively correlated to whole brain volume,^35^ perhaps suggestive that increasing NfL levels were reflective of passive release from brain atrophy during the trial duration.

In Huntington’s disease (HD), CSF NfL was included as an exploratory fluid biomarker during the 15-month Open-Label Extension study of Tominersen, which ultimately remained above baseline measures during the study duration.^37^ In amyotrophic lateral sclerosis (ALS), trials for riluzole and guanabenz did not show any change in serum NfL concentrations, despite guanabenz meeting its primary endpoint in phase 2 trials.^38,39^ However, during phase 1–2 trials for Tofersen for SOD1 ALS CSF NfL levels were included as an exploratory outcome measure and were found to decrease from baseline to day 85, with a continued decrease observed in both CSF and plasma up to day 169.^40^ By phase 3 (VALOR), plasma NfL was used as a pre-specified secondary end point, with concentrations found to reduce over 28 weeks.^8^

Overall, NfL response across AD, HD and ALS trials have been mixed, but it is likely to be relevant that the most successful clinical outcomes, e.g. SOD1 ALS trial, are associated with early significant reductions in NfL. Successfully interrupting neurodegeneration reduces the CSF and plasma pool of NfL, which, given the time taken to translate and release NfL into the extracellular space we observed *in vivo*, is more likely to be explained by a reduction in NfL passive release rather than a downregulation of NfL synthesis.

Conversely, our data show that newly translated NfL takes at least 53–112 days to appear in CSF, therefore NfL values measured after ∼4 months following a therapeutic intervention could reflect a contribution from NfL passive release (neurodegeneration or physiological axonal remodelling) *and/or* contributions from newly translated NfL. The biological relevance of the appearance of newly labelled NfL in CSF is uncertain, but could represent biological recovery or neuroregeneration, and highlights that the pool of NfL in CSF is more dynamic than previously appreciated. The source of CSF NfL is assumed to be central rather than peripheral, since the CNS is in direct communication with the CSF space. Further dynamic labelling studies are going to be critical to understand the relative contribution of passively released versus newly generated NfL across disease states, particularly when the steady state is disrupted through therapeutic intervention. Clinical labelling protocols reflecting the very long turnover of NfL, with long follow-up periods of a year or more, will be required to fully capture NfL kinetics. Translating this SILK assay into plasma remains a considerable technical feat owing to low plasma abundance and anticipated low isotopic enrichment.

Studying NfL kinetics in human neurons *in vitro* provided evidence of rapid NfL translation and its delayed release to the extracellular space in baseline conditions, which mirrored the *in vivo* findings but at an accelerated rate. This kinetic behaviour is consistent with a contribution from controlled or active mechanisms of release of NfL into the extracellular space as opposed to only passive release mechanisms, as has recently been reported in a primary mouse culture model.^41^ While *in vivo* kinetics of NfL were found to be slower compared to tau in the human CNS (half-life of 23 ± 6.4 days),^20^  *in vitro* kinetics of intracellular NfL, with half-lives of 5.06 ± 0.96 days (Ctrl1) and 6.95 ± 2.79 days (Ctrl2), were similar to tau (6.74 ± 0.45 days).^20^ Ultimately, NfL-SILK *in vitro* supported that intracellular protein kinetic events in the brain can be inferred from extracellular compartments.

This study has limitations. The faster turnover rate and extracellular peptide profile observed *in vitro* compared to *in vivo* might be due to faster axonal remodelling, which might be influenced by the comparatively smaller size and lack of myelination compared to *in vivo*. In addition, a faster metabolic rate *in vitro* and/or more resilient proteostasis mechanisms in the fetal-like phenotype of the iPSC-derived neuronal models used may lead to faster clearance rates. The clinical research labelling SILK protocol was not long enough to capture the maximum TTR of NfL, so further studies will be required to ascertain the full kinetic curve of NfL. Secondly, not all participants had evidence of NfL labelling during the 120-day follow-up period (3/9). Since the limit of detection of labelled NfL was close to our measured values (Supplementary Fig. 9), we cannot ascertain whether this is a technical limitation to detecting low levels of physiological NfL release, or if it reflects an inability of neurons to generate new NfL due to more advanced neurodegenerative disease and neuronal loss. Finally, we cannot localize the source of newly labelled NfL within the central or peripheral nervous system. Isoforms with anatomical specificity may be able to address this limitation in future work.

## Conclusions

In summary, we describe a novel method for quantitating the kinetics of NfL *in vitro* and *in vivo*. We show that NfL is rapidly translated in human brain but takes 2–3 months before appearing in human CSF. NfL is likely to have a very long half-life in the human CNS.

## Supplementary Material

fcaf468_Supplementary_Data

## Data Availability

Data are available from the corresponding author on reasonable request.
