# Supplementary material for: Stable isotope labelling kinetics of neurofilament light
Source: Brain Commun. 2025 Dec 17;7(6):fcaf468. doi: 10.1093/braincomms/fcaf468 (PMC12709284; doi:10.1093/braincomms/fcaf468)
Supplement: fcaf468_Supplementary_Data [file fcaf468_supplementary_data.pdf]

Supplemental Online Content

Leckey CA, Giovannucci TA, Coulton JB, et al. Stable isotope labelling kinetics of neurofilament light.

Table of contents

Supplementary Methods

Supplementary Table 1. Cell lines used in this study

Supplementary Table 2. Oligonucleotides used for RT-qPCR

Supplementary Table 3. Antibodies used for immunocytochemistry

Supplementary Table 4. Leucine enrichment data for the TANGLES and NPH SILK cohorts

Supplementary Table 5. Monitored peptides and ion transitions for profiling and quantitation of unlabelled and labelled NfL by IP-MS/MS and yeast enolase for monitoring mass spectrometry performance

Supplementary Table 6. Kinetic measurements in iPSC-derived neuron lysates

Supplementary Table 7. Monitored peptides and ion transitions for profiling and quantitation of unlabelled and labelled APP and MAPT (tau) proteins by IP-MS/MS

Supplementary Table 8. Kinetic measurements in conditioned media from iPSC-neurons

Supplementary Figure 1. Solubility profile of NfL in brain tissue

Supplementary Figure 2. Characterization of the cell lines used in this study

Supplementary Figure 3. Data related to NfL-SILK kinetics *in vitro*

Supplementary Figure 4. Single peptide curves from NfL-SILK in iPSC-derived neurons from three non-degenerative donors

Supplementary Figure 5. Representative chromatograms of labelled NfL detection by peptide-level IP-MS/MS in TANGLES participants

Supplementary Figure 6. NfL kinetics in TANGLES CSF by protein-level IP-MS/MS

Supplementary Figure 7. Participants with detection of labelled NfL in the Control and MCI SILK cohort

Supplementary Figure 8. Detection of labelled NfL in a participant with iNPH 128 days post-labelling

Supplementary Figure 9. Technical characterization of the NfL peptide-level IP-MS/MS assay

Supplementary References

This supplementary material has been provided by the authors to give readers additional information about their work.

Supplementary Methods

Differentiation of induced-pluripotent stem cells (iPSC) into iPSC-derived cortical neurons

Supplementary Table 1. Cell lines used in this study

Cell line	Control/mutation	Sex	Age of onset	Age at biopsy	APOE genotype
Ctrl1 (Sigma-Aldrich)	Cognitively normal SIGi1001-a-1	F	-	20-24	3/4
Ctrl2 (Sigma-Aldrich)	Cognitively normal RBi001-a	M	-	45-49	3/3
Ctrl3 (Dr Tilo Kunath)	Healthy control line	M	-	75-79	3/3

To monitor the successful differentiation into neurons, we isolated RNA and 4% paraformaldehyde-fixed cells on coverslips from cultures at different points during differentiation (usually at or close to day 0 (iPSC), day 50, day 70, day 100, day >100).

Quantitative PCR: RNA was extracted using phenol:chloroform extraction of TriZol (Sigma) and isolated using the Monarch Nucleic Acid Purification Kit according to manufacturer's instructions. RNA concentration was quantified using a NanoDrop spectrophotometer and 1 µg of total RNA was reverse transcribed to cDNA using Superscript IV (Thermo Fisher) and random hexamers. Gene expression was quantified using ~10 ng cDNA template and Power SYBER Green PCR Master Mix (2X) (Thermo Fisher) using an MX300P real time PCR cycler (Agilent). The relative changes in mRNA expression were calculated using the comparative Ct method and were normalised to the housekeeping gene glyceraldehyde-3-phosphate (GAPDH). The list of primers used in this study can be found in **Supplementary Table 2**.

Immunocytochemistry: Coverslips with cells were fixed in 4% paraformaldehyde for 15 minutes and stored in phosphate buffered saline (PBS) until immunostaining was performed. Cells were permeabilized in 0.3% Triton X-100 in PBS for 30 minutes, then blocked in 5% bovine serum albumin (BSA) in PBS for 1 hour. Following three washes in PBS, cells were incubated overnight in primary antibodies (see **Supplementary Table 3**). Cells were washed thrice in PBS and incubated in secondary antibodies (AlexaFluor 488, 568, 594, 647; Thermo Fisher Scientific) for 1 hour, light protected. DAPI was added as a nuclear counterstain at 0.2 ug/ml. Mounted coverslips were imaged using a Zeiss LSM microscope and Leica LAS software.

Supplementary Table 2. Oligonucleotides used for RT-qPCR

Gene name	Protein encoded	Forward Sequence (5'-3')	Reverse Sequence (5'-3')
GAPDH	Glyceraldehyde-3-Phosphate Dehydrogenase	CGCTCTCTGCTCCTCCTGTT	CCATGGTGTCTGAGCGATGT
OCT3/4	Octamer-binding transcription factor 3/4	TTCTGGCGCCGGTTACAGAACCA	GACAACAATGAAAATCTTCAGGAGA
TBR1	T-box brain transcription factor 1	ATCCACAGACCCCCTCACTAG	ATCCACAGACCCCCTCACTAG
TUBB3	Tubulin beta-3 chain	CATGGACAGTGTCCGCTCAG	CAGGCAGTCGCAGTTTTCAC
NEFL	Neurofilament light chain	TGAGGAATGGTTCAAGAG	TGATCGTGTCCTGCATAG

Supplementary Table 3. Antibodies used for immunocytochemistry

**Antigen**	**Provider**	**Catalog number**	**Dilution**
NANOG	Cell Signalling Technologies	4903	1:200
SSEA4	Biolegend	330402	1:200
NEFL	(T.400.5) Thermo Fisher	MA5-14981	1:200
TBR1	Abcam	ab31940	1:500
TUBB3 (TUJ1)	Biolegend	802001	1:1000
Ki67	BD Pharmingen	550609	1:50
MAP2	Abcam	ab5392	1:500
Pax6	Biolegend	901301	1:100

Lactate dehydrogenase (LDH) assay

Media from cultured cells was collected and diluted in LDH Storage Buffer (200 mM Tris-HCl pH 7.3, 10% Glycerol, 1% BSA). The dilution factor was determined by finding the linear range of the LDH positive control following titration. Samples were equilibrated at room temperature before 25 µL of each sample was added to a 96-well opaque-walled assay plate in duplicate wells. The following controls were also set up in duplicate: no-cell control (containing only culture medium) and maximum LDH release control (obtained from cells treated with 10% Triton X-100). To each well, 25 µL of LDH Detection Reagent (25 µL LDH Detection Enzyme and 0.12 µL Reductase Substrate, prepared for all samples) was added before a 1 hour incubation period. Absorbance at 450 nm was measured using a Tecan Spark 10M plate reader.

LDH release was used as a proxy of cell membrane permeability due to cytotoxicity. The results were reported as percentages relative to the positive control following the formula:

LDH release (%) = (Experimental LDH release – Medium background) /

(Experimental LDH release positive control – Medium background)

NfL-SILK assay development and characterization

We previously developed two targeted mass spectrometry assays for NfL; peptide-level immunoprecipitation-tandem mass spectrometry (IP-MS/MS) for targeted quantitation of a NfL Coil 2B peptide, and protein-level immunoprecipitation-mass spectrometry (IP-MS) assay for quantitation of peptides from Coils 1A, 1B, 2B and tail subdomain B of NfL[1]. Both assays were adapted for SILK by addition of monitored ion transitions for $^{13}C_6$-leucine labelled peptides (**Supplementary Table 3**) and used to measure isotopic enrichment of NfL *in vitro* and *in vivo*.

Analysis of NfL was conducted on an Acquity™ I-Class PLUS UPLC coupled to a Xevo™ TQ-XS triple quadrupole mass spectrometer operated in positive electrospray ionization (ESI+) mode (Waters Corporation). Samples were injected onto an Acquity Premier peptide ethylene bridged hybrid (BEH) C18 analytical column (300 Å, 1.7 µm, 2.1 × 50 mm) maintained at 50°C. Initial mobile phase composition was set to 97% A (0.1% FA) and 3% B (ACN, 0.1% FA) at 0.2 mL/min. Chromatographic separation was performed over the following 16-min gradient: initial conditions were held until 0.2 min after which B was linearly increased to 35%

by 11 min. To wash the column, B was increased to 100% over a 1-min linear gradient and held for 1.8-min at an increased flow rate of 0.6 mL/min before returning the system to initial conditions and re-equilibrating the column for 2.2 min. Mass spectrometer settings were as follows: 300°C desolvation temperature, 600 L/h desolvation gas flow, 2.5 kV capillary voltage, 150 L/h cone gas and 0.15 mL/min collision gas.

We assessed the sensitivity of the peptide-level IP method to detect isotopically-labelled NfL using custom TLEIEACR (Coil 2B, [residues 316-323]) AQUA QuantPro peptides spiked into an artificial CSF (aCSF) matrix at different tracer ($^{13}C_6$-leucine peptide) to tracee (unlabelled peptide) ratios (TTR), with unlabelled NfL pitched at the expected average concentration in elderly healthy controls (600 pg/mL)[2]. The assay limit of detection (LoD) and limit of quantitation (LoQ) were calculated by a simple linear regression model of the calibration curve using Excel. Using the following equations:

$$LoD= (3.3*\text{Std error of regression})/\text{slope})$$

$$LoQ = (10*\text{Std error of regression})/\text{slope})$$

the LoD and LoQ were determined to be 0.08% TTR and 0.24% TTR respectively (**Supplementary Figure 9**).

NfL-SILK quantitation

Acquired data was imported into Skyline (v24.1, MacCoss Lab, University of Washington) or MassLynx (v4.2, Waters Corporation) software for peak picking and integration, and QC checks including retention time stability, quantifier/qualifier ion ratios and signal/noise measures. To calculate NfL tracer-to-tracee ratio (TTR), labelled peak areas were divided by unlabelled peak areas and represented as percentages. NfL kinetic curves were plotted in GraphPad Prism. The half-life of NfL was calculated by fitting each peptide-level data into a one-phase decay model. For calculating the average half-life of NfL, the estimated half-lives for single peptides were used. Estimates where a double-sided confidence interval could not be reported were considered unreliable and excluded. Peptides containing two leucine resides in their sequence were normalized following the formula:

$$TTR\_corrected\_for\_2\_leu\_double\_label = TTR/(TTR + 0.5)$$

The fractional synthesis rate (FSR) was calculated using the standard formula:

$$FSR = (Et2 - Et1)NfL/(t2 - t1) / \text{Precursor } E$$

where (Et2 − Et1)NfL/(t2 − t1) was defined as the slope of the linear regression from 3 to 24 days of labelling divided by the leucine enrichment in media (at 50 mol, this equals 1). The fractional clearance rate (FCR) was calculated by fitting the slope of the natural logarithm of the "clearance" portion of the labelled NfL curve (i.e, the chase) according to the formula:

$$FCR = \ln(\text{labelled NfL}/\text{unlabelled NfL}) / (t2 - t1)$$

Half-lives, FSR and FCR estimates are reported rounded to the second decimal place.

For the WashU Tau SILK cohort (cognitively normal and MCI cases), quantitation of endogenous NfL levels was calculated as described in Leckey et al[1] and reported in pg/ml.

For profiling of NfL, peak areas of endogenous peptides were used. The abundance of each peptide was normalized to the total peptide abundance in the sample measured by a colorimetric peptide assay (Thermo Scientific). To aid comparisons between compartments,

peptide abundances were divided by the abundance of a common rod domain peptide detected across compartments [324-331].

Leucine enrichment in plasma

To quantitate labelled leucine enrichment in plasma, a hydrophilic interaction liquid chromatography – tandem mass spectrometry (HILIC-MS/MS) assay was adapted from Prinsen et al[3] to measure $^{13}C_6$-/$^{12}C_6$-leucine ratios, with the following optimisations and adaptations:

Analysis was performed using an Acquity H-Class Ultra Performance Liquid Chromatography (UPLC) system, fitted with an ACQUITY UPLC BEH Amide column (100A; 1.7µm, 2.1 x 50mm) attached to a VanGuard UPLC BEH Amide precolumn (2.1 x 5 mm), which was coupled to a Xevo TQ-S triple quadrupole mass spectrometer operated in positive electrospray ionisation (ESI+) mode (Waters, UK). Chromatographic separation was performed over a 10-minute HILIC gradient using mobile phases A (10 mM ammonium formate in 85% ACN 0.15% FA) and B (10mM ammonium formate in ultrapure Milli-Q water 0.15% FA). The column was primed and equilibrated for 45 minutes in initial conditions (100% A at 0.4 mL/min) and kept at 35˚C. At injection (1 µL/sample), the column was kept in initial conditions for 3 minutes until a linear gradient of increasing %B began. From 3 – 3.1 minutes B was increased to 5.9%, and from 3.1 – 5 minutes was increased to 17.6%, followed by a final increase to 29.4% from 5 – 6 minutes. The flow rate was then increased to 0.6 mL/min and the column re-equilibrated in 100% A for 4 minutes. Mass spectrometer parameters were set as follows: source temperature (150˚C), capillary voltage (1.00 kV), desolvation temperature (550˚C), desolvation gas flow (1000 L/hr) and cone gas flow (150 L/hr).

Ion transitions for $^{12}C_6$-leucine (precursor: 132.102 m/z, product: 86.100 m/z), $^{13}C_6$-leucine (precursor: 138.122 m/z, product: 91.139 m/z) and $^{13}C_6$-,$^{15}N_2$-lysine internal standard (precursor: 155.127 m/z, product: 90.100 m/z) were analysed by multiple reaction monitoring (MRM) and acquired data imported into Skyline (MacCoss Lab, University of Washington) for processing and peak integration. Peak areas were exported into Microsoft Excel and leucine TTR calculated as molar $^{13}C_6$-leucine/$^{12}C_6$-leucine peak area ratios.

For TANGLES, the leucine enrichment in plasma was calculated as the average of leucine enrichment at plateau points (between 6-16 hours of labelling) and reported as percentage (**Supplementary Table 4**). Because labelling in the NPH SILK cohort was variable (dependent on surgery timings), not all subjects showed a plateau in the plasma leucine enrichment. Leucine enrichment data from the two NPH subjects with *ex-vivo* brain biopsies is taken from one subject in the NPH SILK cohort that showed plateau and reported as percentage (**Supplementary Table 4**).

Supplementary Table 4. Leucine enrichment data for the TANGLES and NPH SILK cohorts.

**Participant ID**	**Leucine enrichment in plasma (TTR[a] % at plateau)**
**TANGLES SILK study**	
P01	42.6
P02	41.01
P03	47.57
P04	42.96
P05	55.65
P06	55.06
P07	48.15
P08	41.70
P09	39.44
P10	36.99
**NPH SILK study**	
N01	23.49
N02	23.49
N03	23.49

[a]TTR: tracer-to-tracee ratio.

Supplementary Table 5. Monitored peptides and ion transitions for profiling and quantitation of unlabelled and labelled NfL by IP-MS/MS and yeast enolase for monitoring mass spectrometry performance

Structural domain	Amino acids	Peptide sequence	Precursor ion (m/z)	Precursor charge (z)	Product ion (type)	Product ion (m/z)
Head	31-37	SGYSTAR	371.1799	2	y4+	434.2358
					y5+	597.2991
		SGYSTAR [ISTD][a]	376.1841	2	y4+	444.2440
					y5+	607.3074
Coil 1A	92-100	AQLQDLNDR	536.7727	2	y7+	873.4425
					y5+	632.2998
		AQLQDLNDR [SILK]	539.7828	2	y7+	879.4626
					y5+	638.32
		AQLQDLNDR [SILK]			y7+	879.4626
					y5+	632.2998
		AQLQDLNDR [SILK]	542.7928	2	y7+	885.4827
					y5+	638.32
		AQLQDLNDR [ISTD]	541.7769	2	y7+	883.4507
					y5+	642.3081
	101-107	FASFIER	435.2294	2	y5+	651.3461
					y6+	722.3832
		FASFIER [ISTD]	440.2336	2	y5+	661.3543
					y6+	732.3914
Coil 1B	117-126	VLEAELLVLR	577.8606	2	y8+	942.5619
					y7+	813.5193
		VLEAELLVLR [SILK]	583.8809	2	y8+	948.582
					y7+	819.5394
		VLEAELLVLR [ISTD]	582.8649	2	y8+	952.5701
					y7+	823.5275
	137-144	ALYEQEIR	511.2693	2	y6+	837.4101
					y4+	545.3042
		ALYEQEIR [SILK]	514.2793	2	y6+	837.4101
					y4+	545.3042
		ALYEQEIR [ISTD]	516.2734	2	y6+	847.4184
					y4+	555.3125
	148-157	LAAEDATNEK	531.2591	2	y8+	877.3898
					y6+	677.3101
		LAAEDATNEK [SILK]	534.2692	2	y8+	877.3898
					y6+	677.3101
		LAAEDATNEK [ISTD]	535.2662	2	y8+	535.2662
					y6+	685.3243
	178-185	YEEEVLSR	512.7509	2	y6+	732.3886
					y5+	603.3461
		YEEEVLSR [SILK]	515.761	2	y6+	738.4088
					y5+	609.3662
		YEEEVLSR [ISTD]	517.7551	2	y6+	742.3969
					y5+	613.3543

**Structural domain** (continued)	**Amino acids** (continued)	**Peptide sequence** (continued)	**Precursor ion (m/z)** (continued)	**Precursor charge (z)** (continued)	**Product ion (type)** (continued)	**Product ion (m/z)** (continued)
Coil 2B	284-293	FTVLTESAAK	533.7926	2	y8+	818.4618
					y7+	719.3934
		FTVLTESAAK [SILK]	536.8027	2	y8+	824.4819
					y7+	725.4135
		FTVLTESAAK [ISTD]	537.7997	2	y8+	826.4760
					y7+	727.4076
	316-323	TLEIEACR	496.2475	2	y6+	777.356
					y5+	648.3134
		TLEIEACR [SILK]	499.2576	2	y6+	777.356
					y5+	648.3134
		TLEIEACR [ISTD]	501.2516	2	y6+	787.3642
					y5+	658.3216
	324-331	GMNEALEK	446.2157	2	y6+	703.3621
					y4+	460.2766
		GMNEALEK [SILK]	449.2257	2	y6+	709.3822
					y4+	466.2967
		GMNEALEK [ISTD]	450.2228	2	y6+	711.3763
					y4+	468.2908
Tail Subdomain B	530-540	VEGAGEEQAAK	544.7646	2	y9+	860.4108
					y7+	732.3523
		VEGAGEEQAAK [ISTD]	548.7717	2	y9+	868.4250
					y7+	740.3665
Yeast Enolase (ISTD)	16-28	GNPTVEVELTTEK	708.8645	2	y11++	623.3323
					y8+	948.4884

[a]ISTD: internal standard.

For each NfL peptide, ion transitions are provided for endogenous NfL (unlabelled), $^{13}C_6$-leucine labelled NfL [SILK] and the $^{13}C_6$, ^{15}N-labelled Arg/Lys NfL internal standard [ISTD].

Quantifier and qualifier product ions are listed for each peptide, with the quantifier listed first.

Supplementary Table 6. Kinetics measurements in iPSC-derived neuron lysates

HALF-LIFE

		Ctrl1					Ctrl2			Ctrl3		
**Peptide**	**Amino acids**[a]	**n**[b]**1**	**n2**	**n3**	**n4**	**Mean half-life (d**[c]**) ± SD**[d]	**n1**	**n2**	**Mean half-life (d)**	**n1**	**n2**	**Mean half-life (d)**
AQLQDLNDR	92-100	4.48	6.51	5.07	7.10	5.79 ± 1.22	9.11*	4.44	6.78	NC[f]	NC	NC
ALYEQEIR	137-144	2.75	4.99	3.42	4.87	4.01 ± 1.10	70.45*	6.12	38.29*	15.71*	2.33*	NC
LAAEDATNEK	148-157	2.34	4.91	3.17	5.20	3.91 ± 1.37	26.13*	10.56	18.35*	7.67	3.90*	7.76
FTVLTESAAK	284-293	NC	5.42	NC	5.54	5.48 ± 0.08	10.35	7.73*	9.04	NC	NC	NC
TLEIEACR	316-323	3.73	8.48	NC	6.54	6.25 ± 2.39	25.37*	5.68	15.53*	NC	NC	NC
GMNEALEK	324-331	NC	4.50	NC	5.37	4.93 ± 0.62	5.29	3.78	4.53	NC	NC	NC
	Mean all					**5.06** ± 0.96			**6.95** ± 2.79			**7.76**

FRACTIONAL SYNTHESIS RATE (Intracellular)

**Peptide**	**Amino acids**	**n1**	**n2**	**n3**	**n4**	**Mean FSR**[g] **(%/d) ± SD**	**n1**	**n2**	**Mean FSR (%/d) ± SD**	**n1**	**n2**	**Mean FSR (%/d) ± SD**
AQLQDLNDR	92-100	3.02	1.78	2.01	1.83	2.16 ± 0.58	1.38	1.37	1.37 ± 1.48	NC	NC	NC
ALYEQEIR	137-144	4.55	3.00	3.3	2.82	3.42 ± 0.78	1.84	1.71	1.77 ± 1.65	2.52	2.23	2.38 ± 0.21
LAAEDATNEK	148-157	5.25	3.26	3.27	2.76	3.63 ± 1.10	1.86	1.62	1.74 ± 1.22	2.58	2.17	2.37 ± 0.00
FTVLTESAAK	284-293	NC	2.93	NC	2.79	2.86 ± 0.10	2.20	1.87	2.03 ± 2.06	NC	NC	NC
TLEIEACR	316-323	4.79	2.09	NC	2.36	3.08 ± 1.49	1.80	1.60	1.70 ± 1.30	NC	NC	NC
GMNEALEK	324-331	NC	3.13	NC	1.89	2.51 ± 0.88	1.92	1.89	1.91 ± 1.41	NC	NC	NC
	Mean all					**2.94** ± 0.55			**1.75** ± 0.20			**2.37** ± 0.1

FRACTIONAL CLEARANCE RATE (Intracellular)

**Peptide**	**Amino acids**	**n1**	**n2**	**n3**	**n4**	**Mean FCR**[h] **(%/d) ± SD**	**n1**	**n2**	**Mean FCR (%/d) ± SD**	**n1**	**n2**	**Mean FCR (%/d) ± SD**
AQLQDLNDR	92-100	12.12	7.28	8.39	8.10	8.97 ± 2.15	4.56	2.46	3.51 ± 1.48	NC	NC	NC
ALYEQEIR	137-144	10.60	7.04	7.96	7.17	8.19 ± 1.66	5.10	2.76	3.93 ± 1.65	8.24	10.37	9.31 ± 1.51
LAAEDATNEK	148-157	7.74	7.14	8.18	7.69	7.69 ± 0.43	4.74	3.02	3.88 ± 1.22	7.96	8.34	8.15 ± 0.27

FRACTIONAL CLEARANCE RATE (Intracellular) (continued).

		Ctrl1					Ctrl2			Ctrl3		
**Peptide**	**Amino acids**	**n1**	**n2**	**n3**	**n4**	**Mean FCR (%/d) ± SD**	**n1**	**n2**	**Mean FCR (%/d) ± SD**	**n1**	**n2**	**Mean FCR (%/d) ± SD**
FTVLTESAAK	284-293	NC	7.37	NC	7.87	7.62 ± 0.35	5.85	2.93	4.39 ± 2.06	NC	NC	NC
TLEIEACR	316-323	9.35	7.06	NC	7.27	7.89 ± 1.27	4.35	2.51	3.43 ± 1.30	NC	NC	NC
GMNEALEK	324-331	NC	5.95	NC	5.51	5.73 ± 0.31	3.89	1.89	2.89 ± 1.41	NC	NC	NC
	Mean all					**7.68** ± 1.08			**3.67** ± 0.51			**8.73** ± 0.82

[a]Amino acids in the consensus sequence of NfL as per UniProt entry P07196.

[b]n: Number of biologically independent inductions.

[c]d: days. The half-life estimates were calculated fitting the data into a one-phase decay model.

[d]SD: Standard deviation.

[e]*: Half-life measurements marked with an asterisk (*) were deemed unreliable (the model could not report a double-sided confidence interval) and were not included in the calculation of the average.

[f]NC: Not captured (chromatography peaks did not pass quality control checks and were not included in the analysis).

[g]FSR: Fractional synthesis rate (represented as percentage per day ± SD).

[h]FCR: Fractional clearance rate (represented as percentage per day ± SD).

Supplementary Table 7. Monitored peptides and ion transitions for profiling and quantitation of unlabelled and labelled APP and MAPT (tau) proteins by IP-MS/MS. Related to Figure 2.

**Structural domain**	**Amino acids**	**Peptide sequence**	**Precursor ion (m/z)**	**Precursor charge (z)**	**Product ion (type)**	**Product ion (m/z)**
**MAPT (tau) protein**						
Mid-domain	529-538	TPSLPTPPTR	533.7982	2	y6+	668.3726
					b4+	399.2238
		TPSLPTPPTR [SILK]	536.8083	2	y6+	668.3726
					b4+	405.2439
**Amyloid precursor protein (APP)**						
Soluble APP alpha/beta	461-468	VEAMLNDR	474.2344	2	y6+	719.3505
					y4+	517.2729
		VEAMLNDR [SILK]	477.2444	2	y6+	725.3706
					y4+	523.2930

Supplementary Table 8. Kinetic measurements in conditioned media from iPSC-neurons

FRACTIONAL SYNTHESIS RATE (Extracellular)

	**Ctrl1**						**Ctrl2**			**Ctrl3**		
**Peptide**	**Amino acids**[a]	**n1**[b]	**n2**	**n3**	**n4**	**Mean FSR**[c] **(%/d) ± SD**[d]	**n1**	**n2**	**Mean FSR (%/d) ± SD**	**n1**	**n2**	**Mean FSR (%/d) ± SD**
AQLQDLNDR	92-100	2.41	2.59	NA[e]	1.64	2.21	1.23	0.99	1.11	2.20	1.64	1.92 ± 0.39
ALYEQEIR	137-144	2.70	2.87	NA	1.86	2.48	1.36	0.59	0.98	2.71	2.14	2.42 ± 0.40
LAAEDATNEK	148-157	2.89	2.78	NA	2.05	2.57	1.37	1.04	1.20	2.44	2.22	2.33 ± 0.15
FTVLTESAAK	284-293	3.17	3.48	NA	1.94	2.86	1.21	1.20	1.20	3.63	1.24	2.43 ± 1.69
TLEIEACR	316-323	2.90	3.17	NA	2.54	2.87	1.58	1.23	1.40	3.16	2.19	2.67 ± 0.68
GMNEALEK	324-331	2.93	3.54	NA	2.53	3.00	1.53	1.26	1.39	3.17	2.34	2.75 ± 0.59
	Mean all					**2.75** ± 0.30			**1.21** ± 0.16			**2.42** ± 0.29

FRACTIONAL CLEARANCE RATE (Extracellular)

**Peptide**	**Amino acids**	**n1**	**n2**	**n3**	**n4**	**Mean FCR**[f] **(%/d) ± SD**	**n1**	**n2**	**Mean FCR (%/d) ± SD**	**n1**	**n2**	**Mean FCR (%/d) ± SD**
AQLQDLNDR	92-100	4.74	6.01	NA	4.03	4.93	2.36	0.15	1.26	4.32	1.79	3.06 ± 1.79
ALYEQEIR	137-144	4.80	3.95	NA	9.95	6.23	2.92	0.99	1.96	8.56	0.34	4.45 ± 5.81
LAAEDATNEK	148-157	4.08	4.22	NA	5.38	4.56	3.49	1.10	2.30	5.25	1.07	3.16 ± 2.96
FTVLTESAAK	284-293	2.19	3.29	NA	4.80	3.43	1.76	1.07	1.42	5.58	2.92	4.25 ± 1.88
TLEIEACR	316-323	4.05	5.22	NA	6.00	5.09	3.04	1.79	2.42	6.92	3.12	5.02 ± 2.69
GMNEALEK	324-331	4.52	5.20	NA	5.93	5.22	3.13	1.31	2.22	7.61	5.54	6.58 ± 1.46
	Mean all					**4.90** ± 0.92			**1.92** ± 0.48			**4.42** ± 1.30

[a]Amino acids in the consensus sequence of NfL as per UniProt entry P07196.

[b]n: Number of the biologically independent induction.

[C]FSR: Fractional synthesis rate (represented as percentage per day ± SD).

[d]SD: Standard deviation.

[e]NA: Not available

[f]FCR: Fractional clearance rate (represented as percentage per day ± SD).

Supplementary Figure 1. Solubility profile of NfL in brain tissue

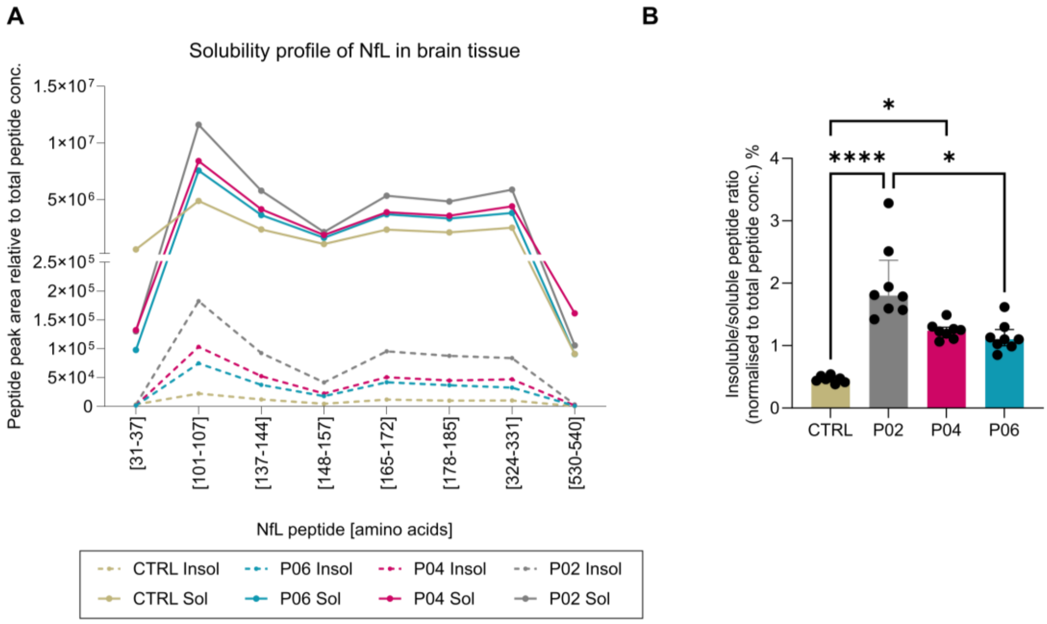

A) Peptide recovery across the NfL sequence after immunoprecipitation normalised to total peptide concentration in the samples. Recovery of NfL was lower in insoluble compared to soluble fractions. Peptide [31-37] abundance was higher in CTRL compared to P02, P04 and P06. **B)** The peptide abundances normalised to total peptide content in the sarkosyl-insoluble fraction ("insoluble") were divided by their abundance in the sarkosyl-soluble ("soluble") fractions to calculate the ratio of insoluble NfL peptides across cases, shown as a percentage (%). Each dot represents a peptide ratio (including peptides within residues [31-37], [101-107], [137-144], [148-157], [165-172], [178-185], [324-331], [530-540] of the NfL amino acid sequence). Results are displayed as median ± IQR. A Kruskal-Wallis test was conducted to compare the ratio between samples (n = eight peptide ratios). There was a significant difference across the groups ($H(3) = 25.61$, $p < 0.0001$). Post-hoc Dunn's multiple comparison's test revealed that P02 (median = 1.80, IQR = 2.37 - 1.58) and P04 (median = 1.24, IQR = 1.29 - 1.13) had significantly higher insoluble to soluble NfL peptide ratios than CTRL (median = 0.47, IQR = 0.51 - 0.42). P02 had significantly higher ratios than P06 (median = 1.10, IQR = 1.26 - 1.00). * < 0.05; **p≤ 0.01; ****p≤ 0.0001. Abbreviations: CTRL, healthy control; Insol, Insoluble; Sol, Soluble.

Supplementary Figure 2. Characterization of the cell lines used in this study

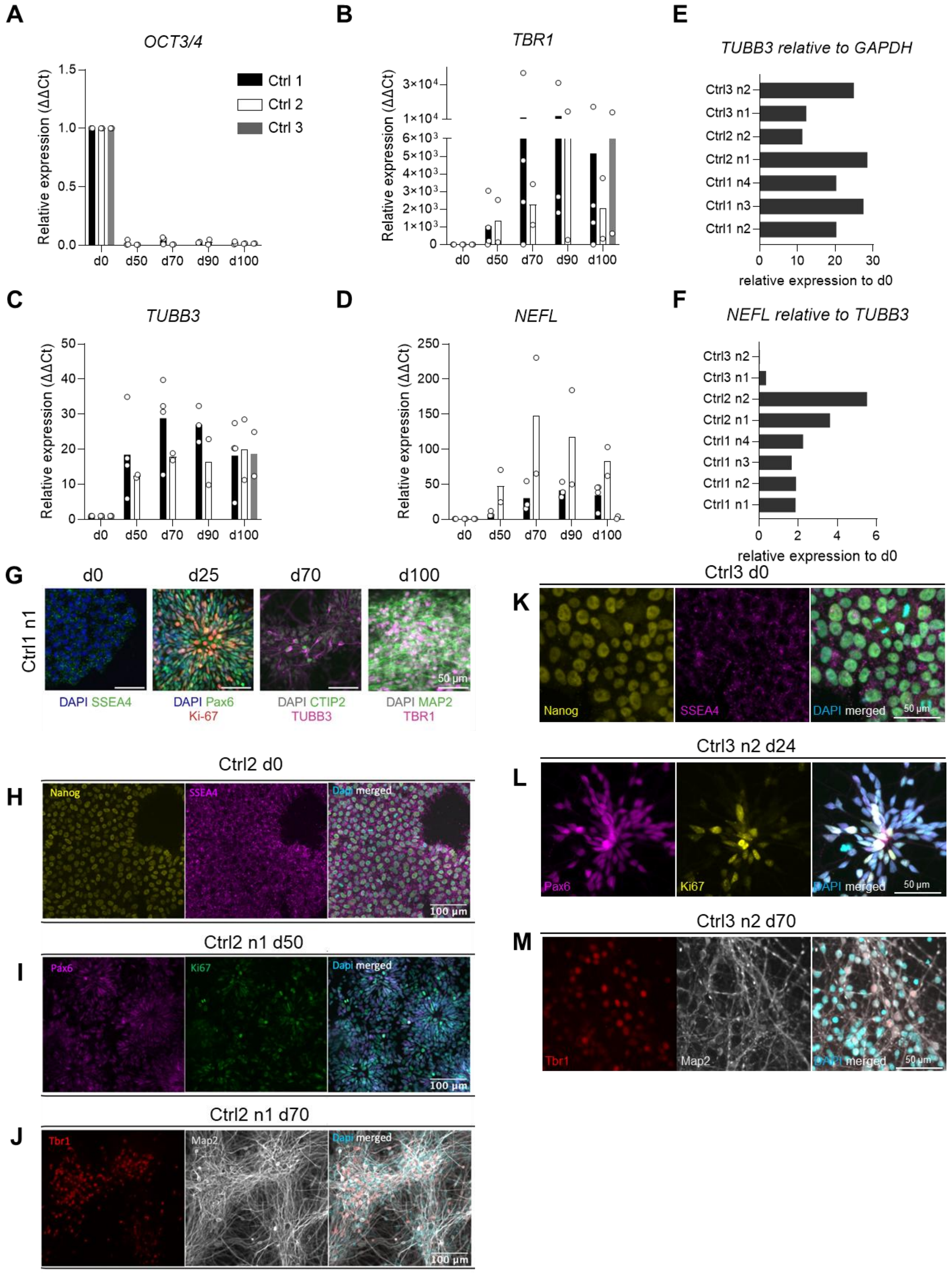

A) Relative levels of the pluripotency gene Octamer-binding transcription factor 3/4 (*OCT3/4*), **B)** T-box brain transcription factor 1 (*TBR1*), **C)** Beta III tubulin (*TUBB3*), **D)** Neurofilament light (*NEFL)* in the different iPSC lines used. Ctrl1 and Ctrl2 include data at days-in-vitro (d) d0, d50, d70-72, d90, d95-103. Ctrl3 include data at d0 and d100 only (due to a lower yield of

neurons, only d100 was prioritised for this analysis). Bars represent the mean. Each open circle represents an independent neuronal induction. Ctrl 1 n=3-4; Ctrl 2 n=2; Ctrl 3 n=2. **E)** *TUBB3* expression relative to the housekeeping gene *GAPDH* at d100-103 in independent inductions used in the study. **F)** *NEFL* expression relative to *TUBB3* at d95-100 in the independent inductions used in the study. **G)** Widefield fluorescence microscopy images of Ctrl1 iPSC stained for the pluripotency markers Nanog and Stage-specific embryonic-antigen 4 (SSEA4) (d0); paired box 6 (Pax6) and Ki67 demonstrating conversion into actively dividing Neural Precursor Cells (NPCs) at 25 days-in-vitro (d25); the deep-layer neuronal marker COUP-TF-interacting protein 2 (CTIP2) and the pan neuronal microtubule-associated protein 2 (MAP2) at d70; the upper-layer neuronal marker T-box brain transcription factor 1 (TBR1) and MAP2 at d100. Scale bar = 50 µm. Ctrl2 n1 cells stained for **H)** Nanog and SSEA4 in iPSCs (d0); **I)** Pax6 and Ki67 at d50; **J)** TBR1 and MAP2 at d70. Scale bar = 100 µm. Ctrl3 cells n3 stained for **K)** Nanog and SSEA4 in iPSCs (d0); **L)** Pax6 and Ki67 at d24; **M)** TBR1 and MAP2 at d70. Scale bar = 50 µm. In all immunostainings, DAPI was used as a nuclear counterstain and is only shown in the merged images.

Supplementary Figure 3. Data related to NfL-SILK kinetics *in vitro*

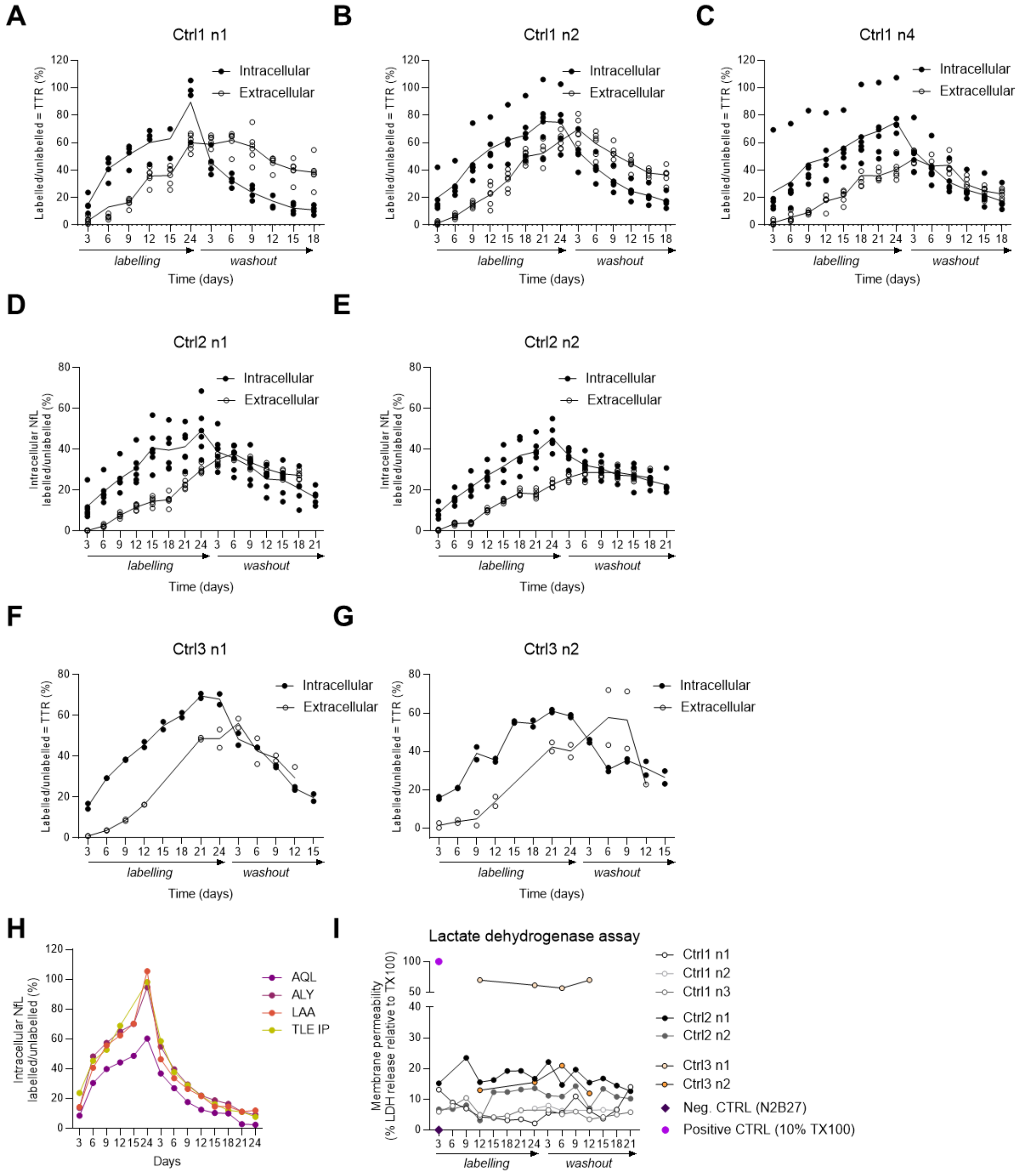

Kinetics in cell lysate ("intracellular") and matched cell media ("extracellular") in three independent inductions of Ctrl1 **(A, B, C)**, two inductions of Ctrl2 **(D, E)** and two inductions of Ctrl3 **(F, G)** cells; related to Figure 2. Each datapoint represents a proteotypic NfL peptide (n=6 peptides). Filled black circles = intracellular datapoints; white circles with black border = extracellular datapoints. **H)** Kinetic curves of proteotypic peptides in NfL (depicted by the first three amino acids of the peptide in 1-letter code) compared to the kinetic profile obtained when performing peptide-level IP with anti-TLEIEACR antibodies ("TLE IP") in Ctrl1 cells (induction n1). **I)** Lactate dehydrogenase release in cell media was measured to assess the levels of cell cytotoxicity in the cultures over the course of the labelling and washout periods of the

experiments. N2B27 only was used as negative control. Cells treated with 10% Triton X-100 were used as positive control. Data are shown as measured LDH release divided by maximum LDH release (elicited by treatment of the positive control sample with 10% Triton X-100 [TX100]).

Supplementary Figure 4. Single peptide curves from NfL-SILK in iPSC-derived neurons from three non-degenerative donors

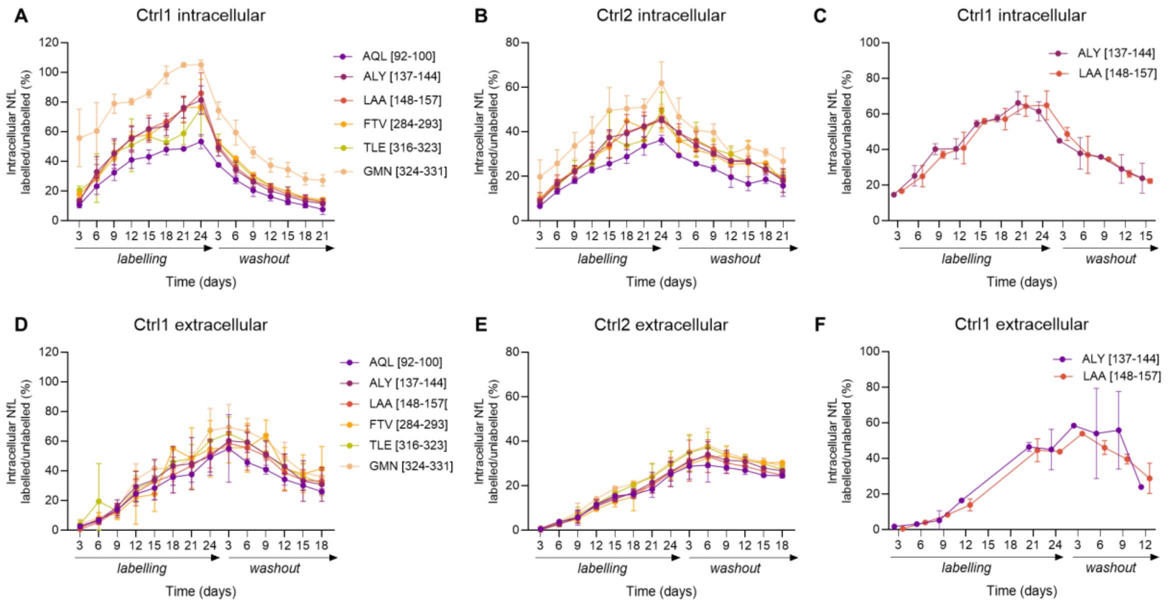

A) Intracellular Ctrl1 results (n=2-4 independent inductions). **B)** Intracellular Ctrl2 results (n=2 independent inductions). **C)** Intracellular Ctrl3 intracellular results (n=2 independent inductions). **D)** Extracellular Ctrl1 results (n=3 independent inductions). **E)** Extracellular Ctrl2 results (n=2 independent inductions). **F)** Extracellular Ctrl3 Extracellular results (n=2 independent inductions). Datapoints represent the mean tracer-to-tracee ratio (TTR) of the peptides at any given timepoint from all inductions ± SD. Peptides are depicted by the first three amino acids of the peptide in 1-letter code, followed by the peptides' first and last residues within the NfL amino acid sequence in brackets. Data related to Figure 2.

Supplementary Figure 5. Representative chromatograms of labelled NfL detection by peptide-level IP-MS/MS in TANGLES participants

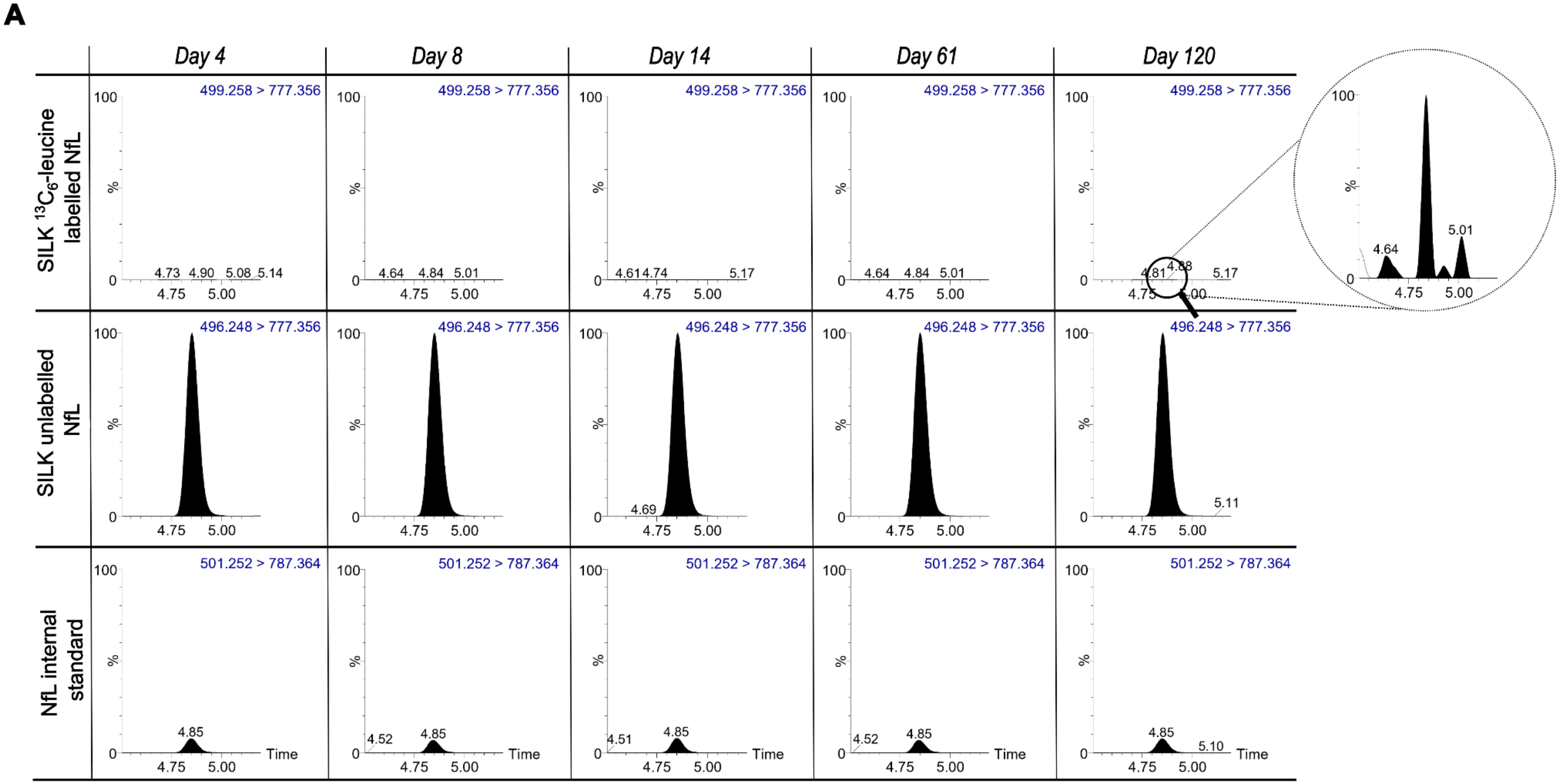

B

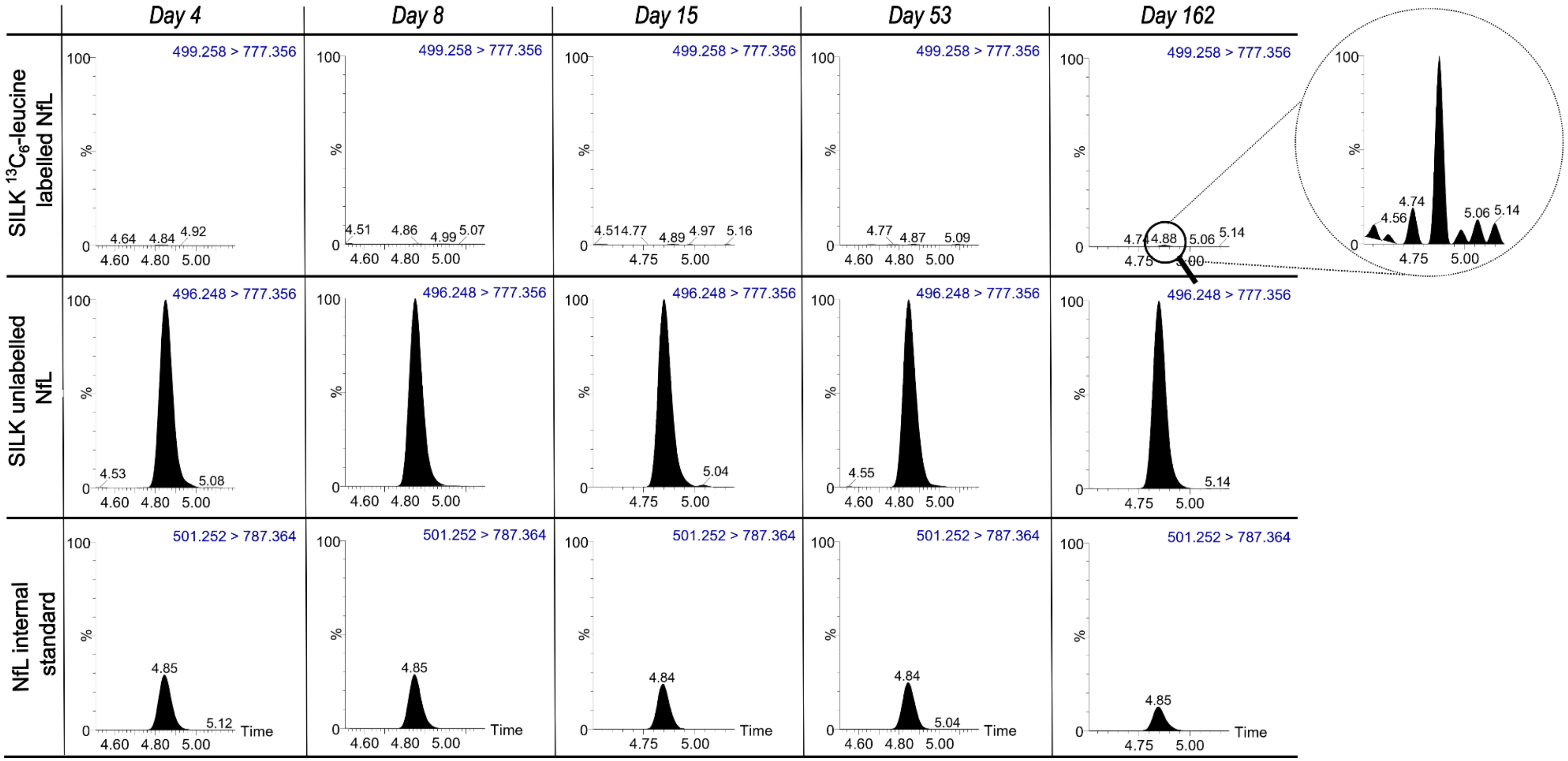

Multiple reaction monitoring (MRM) chromatograms are shown for relative detection of SILK labelled NfL, unlabelled NfL and heavy-labelled internal standard in CSF from two TANGLES participant across the SILK study chase by peptide-level IP-MS/MS. **A)** Participant P06, with labelled NfL detected at chase day 120 (0.12% TTR) and **B)** participant P08, with labelled NfL detected at chase day 162 (0.36% TTR). x-axes represent retention time ("Time"); y-axes represent signal intensity relative to the total ion count (TIC), which represents the intensity of all the masses detected in the channel, shown in percentage (%). Abbreviations: IP-MS/MS, immunoprecipitation – tandem mass spectrometry; SILK, stable isotope labelling kinetics; NfL, neurofilament light chain.

Supplementary Figure 6. NfL kinetics in TANGLES CSF by protein-level IP-MS/MS

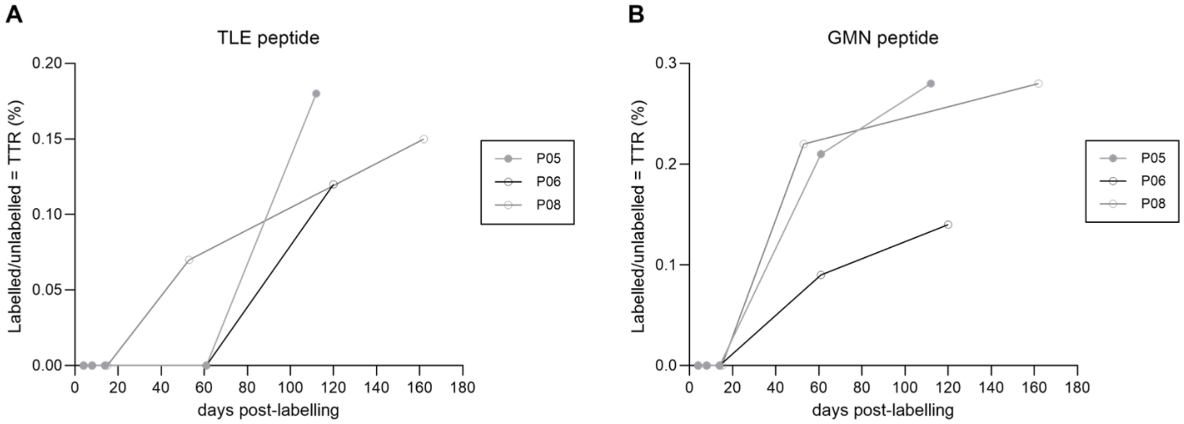

Additional NfL kinetics curves are provided for two peptides of the NfL Coil 2B domain by protein-level IP-MS/MS analysis of the UCL TANGLES cohort. **A)** Kinetics for the TLEIEACR peptide ("TLE"; [316-323]), the same peptide enriched for and detected by the peptide-level IP-MS/MS method, and **B)** kinetics for the GMNEALEK ("GMN"; [324-331]) peptide. As for the peptide-level IP-MS/MS analysis presented in main text Figure 3C, SILK labelled NfL is only detected at the earliest by LP 4 or LP 5 for TANGLES participants P05 (day 61), P06 (day 61) and P08 (day 53), with maximum enrichments by LP 5 of 0.12 - 0.28% for both TLE and GMN peptides. Abbreviations: IP-MS/MS, immunoprecipitation – tandem mass spectrometry; TTR, tracer-to-tracee ratio.

Supplementary Figure 7. Participants with detection of labelled NfL in the WashU Control and MCI cohort

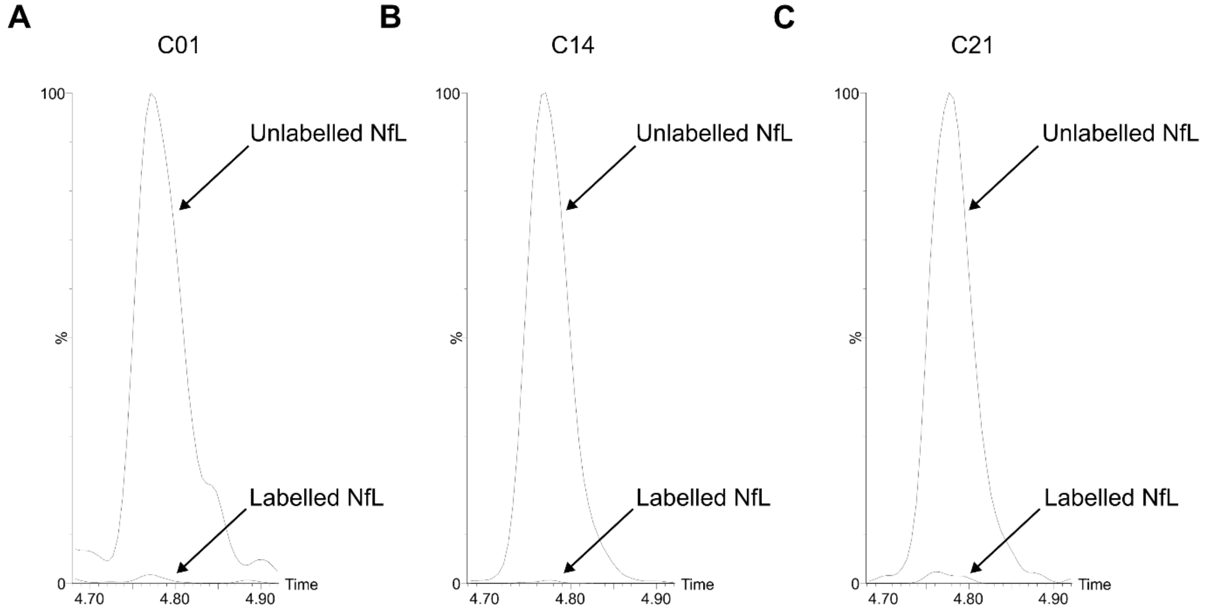

Representative chromatograms for detection of labelled NfL at lumbar puncture visit 5 for three participants from the Control and MCI SILK (WashU) cohort; **A)** participant C01 (CDR = 0.5, CSF Aβ NA), **B)** participant C14 (CDR = 0, CSF Aβ-) and **C)** participant C21 (CDR = 0, CSF Aβ-). Chromatograms shown are overlays of the $^{13}C_6$-leucine labelled NfL monitored ion transition (499.258>777.356 m/z), relative to the unlabelled ($^{12}C_6$-leucine) NfL monitored transition (496.248>777.364 m/z). x-axes represent retention time ("Time") in minutes; y-axes represent signal intensity relative to the total ion count (TIC), which represents the intensity of all the masses detected in the channel, shown in percentage (%).

Supplementary Figure 8. Detection of SILK labelled NfL in a participant with iNPH 128 days post-labelling

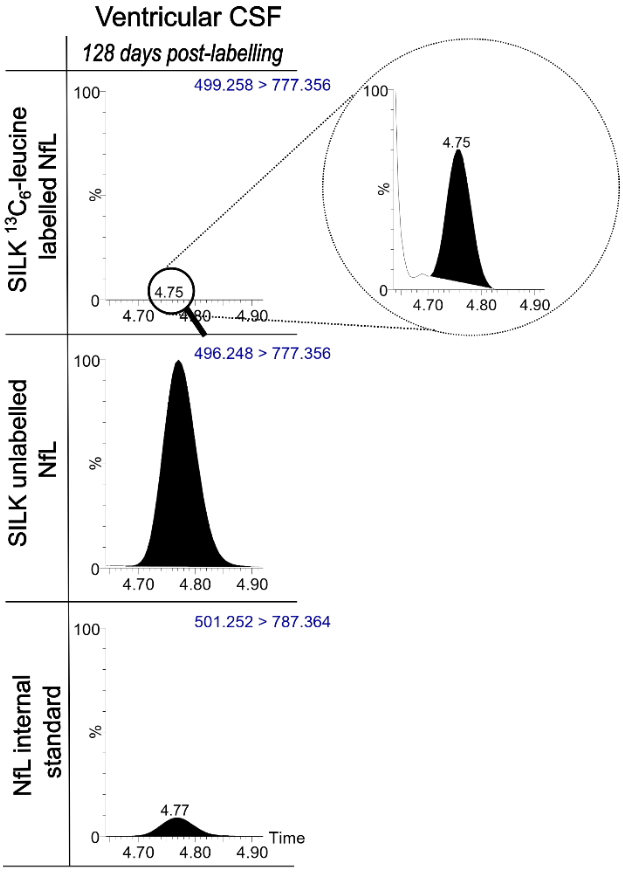

Multiple reaction monitoring (MRM) chromatograms showing the relative detection of labelled and unlabelled NfL, and the heavy labelled internal standard, at day 128 post-labelling in ventricular CSF from a patient with suspected iNPH (n = 1). Peaks shaded and with a stated retention time for the apex of each peak are as detected and processed during peak integration in MassLynx software (v4.2, Waters Corporation). Abbreviations: iNPH, idiopathic normal pressure hydrocephalus; NfL: neurofilament light chain; SILK, stable isotope labelling kinetics.

Supplementary Figure 9. Technical characterization of the NfL peptide-level IP-MS/MS assay

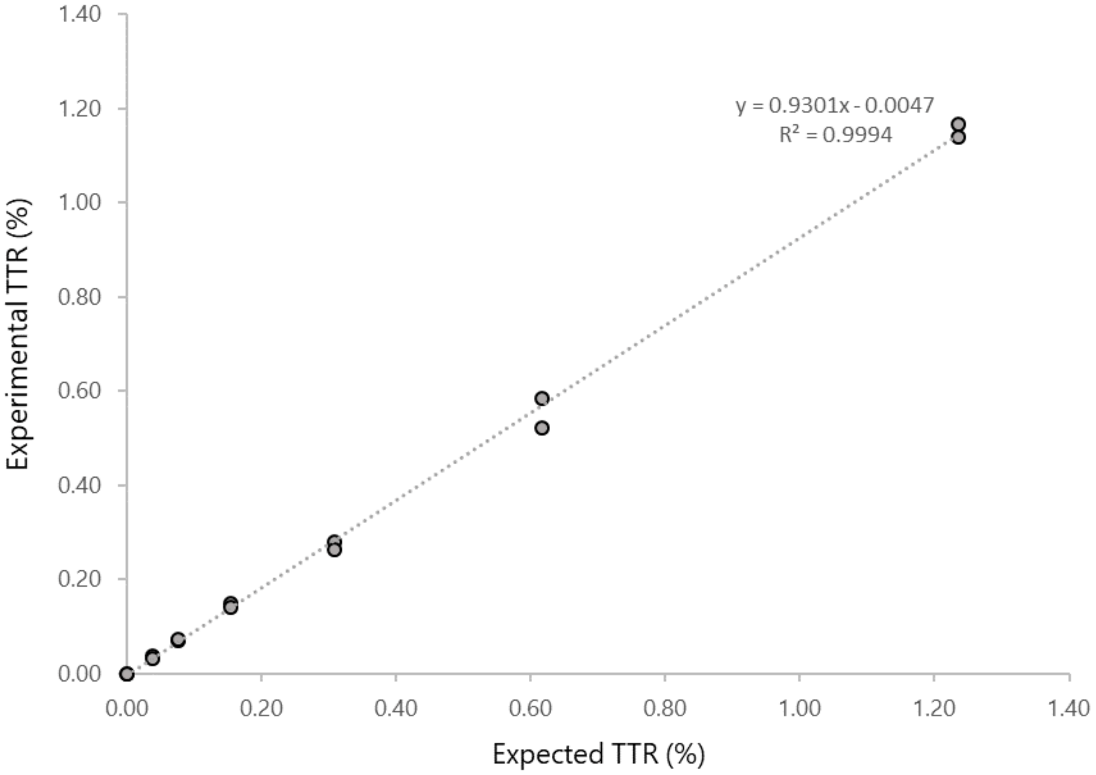

Custom AQUA QuantPro peptides for the Coil 2B peptide (TLEIEACR; [316-323]) were spiked into artificial CSF (aCSF) to assess the sensitivity of the assay as tracer-to-tracee ratio percentage (TTR %), where unlabelled NfL was pitched at 600 pg/mL. From characterization, limit of detection (LoD) and limit of quantitation (LoQ) for SILK TTR% in aCSF was determined to be 0.08% and 0.24% respectively. Datapoints represent technical duplicates. Simple linear regression was used to test if the expected (spiked) TTR significantly predicted the experimental (measured) TTR. The fitted regression model was y=0.9301x-0.0047. The overall regression was statistically significant (R^2 = 0.9994, F(1,5) = 8633.5782, p = 2.74E-09).

Supplementary References

1. Leckey CA, Coulton JB, Giovannucci TA, et al. CSF neurofilament light chain profiling and quantitation in neurological diseases. *Brain Commun.* 2024;6(3):fcae132.
2. Paterson RW, Slattery CF, Poole T, et al. Cerebrospinal fluid in the differential diagnosis of Alzheimer's disease: clinical utility of an extended panel of biomarkers in a specialist cognitive clinic. *Alzheimers Res Ther.* 2018;10(1):32.
3. Prinsen H, Schiebergen-Bronkhorst BGM, Roeleveld MW, et al. Rapid quantification of underivatized amino acids in plasma by hydrophilic interaction liquid chromatography (HILIC) coupled with tandem mass-spectrometry. *J Inherit Metab Dis.* 2016;39(5):651-660.
